# Neutrophils: Heterogeneity, Biology, Diseases, and Therapeutic Strategies

**DOI:** 10.1002/mco2.71104

**Published:** 2026-10-31

**Authors:** Yibing Zhang, Siwen Wu, Yusi Wang, Die Hu, Li Yang

**Affiliations:** ^1^ Department of Biotherapy Cancer Center and State Key Laboratory of Biotherapy West China Hospital Sichuan University Chengdu China

**Keywords:** heterogeneity, neutrophils, neutrophil‐targeted therapy, neutrophil‐based delivery systems, precision drug delivery

## Abstract

Neutrophils are the most abundant circulating leukocytes and are essential components of innate immunity, contributing to host defense, inflammation, tissue remodeling, and disease progression. Increasing evidence has revealed substantial neutrophil heterogeneity and functional plasticity, which enable distinct neutrophil populations to exert protective or pathogenic effects depending on the tissue and disease context. These properties have attracted growing interest in neutrophils as therapeutic targets and as vehicles for targeted drug delivery. This review summarizes the biological characteristics and functional heterogeneity of neutrophils, with particular emphasis on their recruitment, phagocytosis, oxidative responses, extracellular trap formation, and interactions with the tissue microenvironment. We further discuss the diverse roles of neutrophils in cancer, inflammatory diseases, infectious diseases, and other pathological conditions, highlighting their context‐dependent functions. Building on these insights, we review current and emerging therapeutic strategies, ranging from broad neutrophil modulation to precision targeting and neutrophil‐based drug delivery. Living neutrophil carriers, neutrophil membrane‐coated nanoparticles, and neutrophil‐derived vesicles are comparatively evaluated in terms of preparation, cargo loading, targeting properties, advantages, limitations, and translational potential. Finally, we discuss key challenges in the development and clinical translation of neutrophil‐based therapies, including manufacturing, quality control, safety, and therapeutic precision, and highlight future opportunities for their clinical application.

## Graphical abstract

Neutrophils exhibit remarkable functional plasticity and play dual roles in tissue protection and disease progression. This review summarizes their key biological functions and highlights the shift from conventional broad‐spectrum interventions to neutrophil‐based drug delivery systems. We further discuss recent advances and future perspectives in precision immunotherapy leveraging engineered neutrophil platforms.

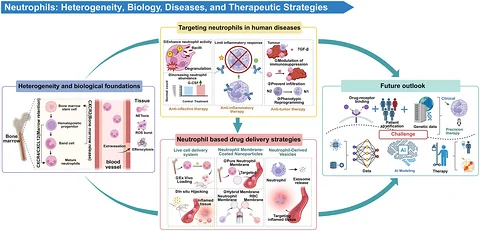

## 1 Introduction

Neutrophils, also known as polymorphonuclear leukocytes (PMNs), are the most abundant leukocyte population in human circulation and serve as the first responders to tissue injury and infection. Beyond their classical antimicrobial functions, emerging evidence from single‐cell and spatial profiling studies has revealed substantial heterogeneity and functional plasticity within neutrophil populations, enabling their adaptation to diverse physiological and pathological environments. These characteristics allow neutrophils to actively migrate toward inflammatory and malignant lesions, making them attractive targets for therapeutic intervention [1, 2].

However, the therapeutic exploitation of neutrophils remains challenging. In many diseases, including cancer, autoimmune disorders, and chronic inflammation, neutrophils undergo disease‐associated reprogramming and contribute to pathological progression through excessive production of inflammatory mediators, reactive oxygen species (ROS), and neutrophil extracellular traps (NETs) [3]. Consequently, conventional strategies aimed at broadly suppressing neutrophil recruitment or activity have shown limited clinical potential because they may simultaneously impair essential host defense functions and disrupt physiological immune homeostasis. Therefore, a major challenge in neutrophil‐based therapy is achieving selective modulation of pathological neutrophil responses while preserving their beneficial immune functions.

Despite significant progress in nanomedicine over recent decades, the clinical translation of synthetic nanoparticles (NPs) remains hindered by substantial physiological barriers [4]. Conventional nanomedicines frequently fail to penetrate biological membranes such as the blood–brain barrier (BBB) or the high interstitial pressure environment characteristic of solid tumors. Additionally, rapid clearance by the mononuclear phagocyte system and instability within the serum environment often lead to suboptimal accumulation at the target site [5]. While surface modifications such as PEGylation have been utilized to prolong circulation, the associated risks of immunogenicity and anti‐PEG antibody responses limit their long‐term efficacy. Consequently, there is an urgent need for endogenous delivery systems capable of active targeting, barrier penetration, and adaptation to complex pathological microenvironments. Neutrophils possess several intrinsic properties that provide unique solutions to these challenges. Their natural ability to sense chemotactic signals, cross biological barriers, and accumulate at sites of inflammation or tumor growth enables them to function as endogenous delivery vehicles [6]. By harnessing these characteristics, neutrophil‐based delivery platforms show promise in addressing the limited targeting specificity and tissue accessibility of conventional nanomedicines. This transition from modulating neutrophil functions to using neutrophils as drug carriers reflects a shift from broad functional targeting toward targeted drug delivery. Current neutrophil‐based therapeutic strategies can therefore be divided into two complementary directions: targeted modulation of endogenous neutrophil functions and engineering of neutrophil‐derived or neutrophil‐inspired delivery systems [7, 8]. The former aims to regulate pathological neutrophil activation, recruitment, or effector functions, whereas the latter exploits neutrophil‐mediated homing and biological adaptability to achieve site‐specific delivery of therapeutic cargos. Understanding the advantages and limitations of these two approaches is essential for developing safer and more effective neutrophil‐based interventions.

In this review, we summarize the heterogeneity, biological functions, disease‐related roles, and therapeutic strategies of neutrophils. We first discuss their key biological properties and functional characteristics, followed by their diverse roles in cancer, inflammatory diseases, infectious diseases, and other pathological conditions. We then review current neutrophil‐based therapeutic strategies, including neutrophil modulation and neutrophil‐based drug delivery systems, and discuss their applications, limitations, and prospects for clinical translation.

## 2 Neutrophil Heterogeneity and Biological Foundations

Neutrophils exhibit substantial heterogeneity and functional plasticity, which are shaped by their unique developmental trajectory and physiological functions. This section first reviews the maturation of neutrophils from hematopoietic stem cells in the bone marrow and the biological processes underlying their functional diversity and homeostasis within the circulation. We then summarize key neutrophil properties, including rapid recruitment, chemotaxis, extravasation, phagocytosis, and tissue infiltration, which enable neutrophils to efficiently sense and respond to inflammatory signals [9]. These intrinsic characteristics allow neutrophils to rapidly migrate across physiological barriers and accumulate at pathological sites, including inflamed and tumor tissues [10]. Understanding these biological foundations provides an essential basis for elucidating the context‐dependent roles of neutrophils in disease and developing neutrophil‐targeted therapeutic strategies.

### 2.1 Neutrophil Development and Heterogeneity

Neutrophil development and heterogeneity provide a fundamental framework for understanding the diverse functions of these cells in health and disease. This section first outlines the developmental and maturation processes of neutrophils, followed by a discussion of their phenotypic and functional heterogeneity across different physiological and pathological contexts. Neutrophils are the most abundant leukocyte population in the human circulation and represent highly plastic innate immune cells with exceptional capabilities in migration, environmental sensing, and immune regulation. Increasing evidence indicates that neutrophils are not merely short‐lived antimicrobial effector cells but rather a heterogeneous and dynamically regulated population comprising distinct maturation states and functional subsets that adapt to diverse microenvironments [11, 12, 13, 14, 15, 16]. This heterogeneity arises from differences in developmental stage, tissue localization, activation state, and exposure to disease‐associated signals, which can substantially influence neutrophil functions and therapeutic responses. As illustrated in Figure 1A, the evolution of neutrophil research has progressively expanded our understanding of these cells from a relatively uniform population to a spectrum of phenotypically and functionally distinct states, providing an important biological basis for understanding their roles in disease and developing neutrophil‐targeted therapeutic strategies.

**FIGURE 1:**
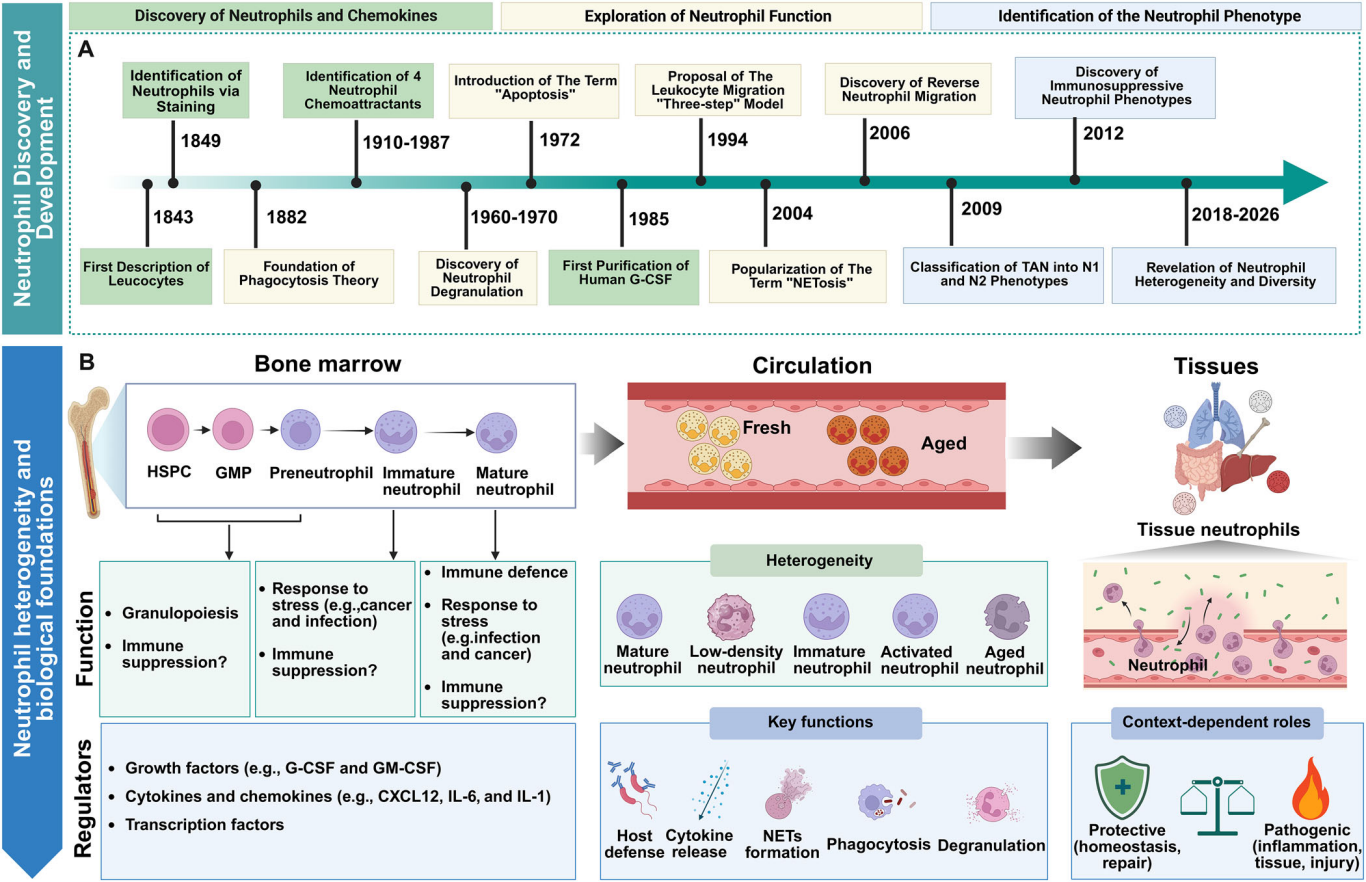
Overview of neutrophil development, heterogeneity, and context‐dependent functions. (A) An overview of milestones in neutrophil development. (B) Neutrophil development, heterogeneity, and context‐dependent functions.

Neutrophil development originates from hematopoietic stem and progenitor cells (HSPCs) in the bone marrow and proceeds through sequential progenitor and maturation stages, including granulocyte–monocyte progenitors (GMPs), preneutrophils, immature neutrophils, and mature neutrophils. This process is tightly regulated by hematopoietic growth factors, cytokines, chemokines, and transcriptional programs, with G‐CSF and GM‐CSF serving as major regulators of granulopoiesis [17]. During maturation, neutrophils undergo progressive changes in morphology, granule composition, surface receptor expression, and functional capacity, establishing the biological foundation for their subsequent responses in the circulation and tissues (Figure 1B). Following maturation, neutrophils enter the circulation, where they exist as a dynamic and heterogeneous population rather than a uniform cell type. Distinct states, including mature, immature, low‐density, activated, and aged neutrophils, exhibit differences in surface phenotype, transcriptional programs, metabolic activity, and effector functions. Recent single‐cell transcriptomic and multiomic studies have further revealed substantial diversity among circulating neutrophils, challenging the conventional view of neutrophils as a homogeneous population. Importantly, these states are not necessarily stable or mutually exclusive subsets. Instead, neutrophils can undergo phenotypic and functional transitions in response to inflammatory signals, systemic conditions, and tissue‐derived cues, highlighting their considerable plasticity. Upon appropriate chemotactic stimulation, circulating neutrophils migrate across the vascular endothelium and accumulate in peripheral tissues. Within different tissue microenvironments, neutrophils can further adapt their phenotype and function. Their major effector activities, including phagocytosis, degranulation, cytokine release, and NET formation, contribute to host defense and tissue homeostasis, but excessive or dysregulated activation can also promote inflammation and tissue injury. Thus, the biological consequences of neutrophil activation are strongly context dependent, with neutrophils potentially exerting protective or pathogenic effects depending on the local microenvironment and disease state.

Neutrophil function is also influenced by interconnected cell death and stress‐response programs, including apoptosis, NETosis, pyroptosis, and ferroptosis. Shared signals such as excessive ROS, metabolic stress, mitochondrial dysfunction, and inflammatory activation can alter neutrophil survival, activation, and effector responses [18]. Rather than representing isolated processes, these pathways form an interconnected regulatory network that contributes to the functional plasticity of neutrophils and provides potential targets for therapeutic intervention [7]. Collectively, neutrophil heterogeneity arises from the interaction between developmental maturation, circulating cellular states, and tissue‐specific environmental cues. Their rapid mobilization, chemotactic migration, environmental responsiveness, and ability to cross vascular and tissue barriers further distinguish neutrophils from conventional drug carriers. These properties provide the biological basis for two complementary therapeutic directions: selective modulation of pathogenic neutrophil populations and exploitation of neutrophils as cellular carriers for targeted drug delivery.

### 2.2 Neutrophil Biology and Functional Plasticity

Neutrophils employ multiple coordinated mechanisms to provide a rapid first‐line defense against invading pathogens, integrating intracellular and extracellular antimicrobial processes (Figure 2). This section summarizes the major antimicrobial mechanisms of neutrophils, including chemotaxis and phagocytosis, intracellular pathogen killing through respiratory burst and degranulation, and extracellular pathogen capture through NETs. Following directional migration toward inflammatory foci, neutrophils recognize and engulf pathogens into specialized phagosomes, where microbial killing is mediated by the synergistic actions of ROS generated during the respiratory burst and proteolytic enzymes and antimicrobial peptides released through degranulation [19]. In addition to these intracellular mechanisms, neutrophils can release NETs, web‐like structures composed primarily of a DNA‐histone backbone decorated with antimicrobial proteins, which facilitate the extracellular capture and neutralization of pathogens [19]. Together, these complementary mechanisms enable neutrophils to detect, sequester, and eliminate invading microorganisms through both intracellular and extracellular strategies, underscoring their central role in innate host defense [20].

**FIGURE 2:**
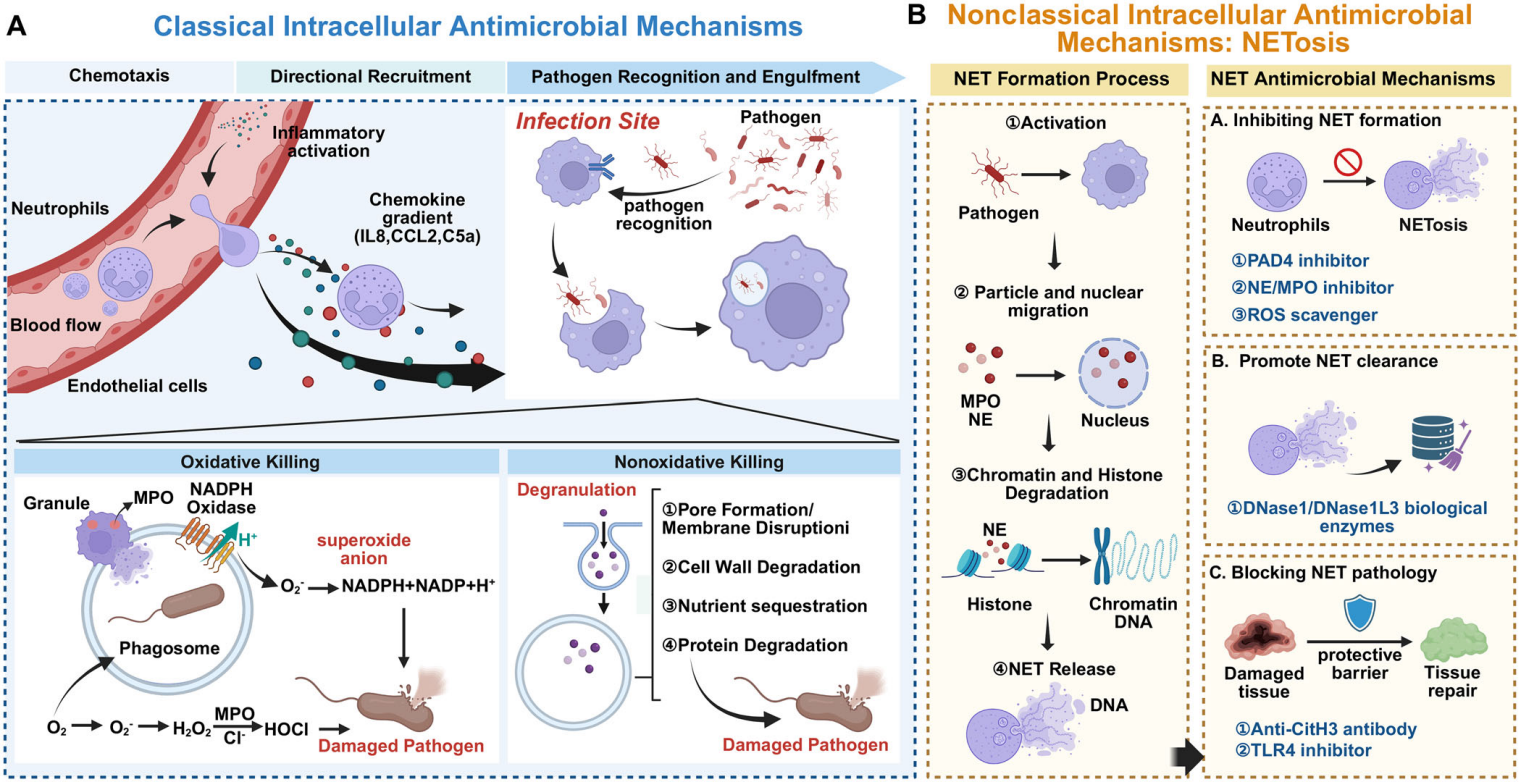
Classical and nonclassical antimicrobial mechanisms of neutrophils. (A) Classical intracellular killing, including migration, phagocytosis, and elimination via ROS‑dependent and nonoxidative (degranulation/protease) mechanisms. (B) NETosis, from activation and chromatin decondensation to NET release. Potential anti‑NET therapeutic strategies are indicated.

#### 2.2.1 Classical Intracellular Antimicrobial Mechanisms

Neutrophils eliminate invading pathogens through a coordinated intracellular defense process (Figure 2A). This process involves three major steps: chemotaxis and directional recruitment to sites of infection, pathogen recognition and engulfment through phagocytosis, and respiratory burst‐mediated oxidative killing within phagosomes. Together, these processes enable neutrophils to rapidly capture and eliminate invading pathogens.

##### 2.2.1.1 Chemotaxis and Directional Recruitment

The recruitment of neutrophils from the circulation to inflammatory sites represents a fundamental feature that enables their potential application as targeted therapeutic carriers [21]. This process is initiated by tissue‐resident sentinel cells, including macrophages and mast cells, which recognize pathogen‐associated molecular patterns (PAMPs) or damage‐associated molecular patterns (DAMPs) through pattern recognition receptors (PRRs) [22]. Subsequently, inflammatory mediators such as chemokines, cytokines, and lipid mediators activate local endothelial cells, inducing the expression of adhesion molecules and establishing chemotactic gradients that guide neutrophil migration toward pathological sites.

Neutrophil recruitment involves a coordinated cascade of vascular interactions, including rolling, adhesion, crawling, and transendothelial migration. Activated endothelial cells upregulate selectins and immunoglobulin superfamily adhesion molecules, allowing circulating neutrophils to be captured and subsequently stabilized through integrin‐mediated interactions, particularly between β2 integrins (LFA‐1 and Mac‐1) and endothelial ligands such as ICAM‐1 [23, 24]. These interactions enable neutrophils to firmly adhere to the vascular wall and migrate across endothelial barriers into surrounding tissues. After extravasation, neutrophils utilize chemokine‐guided amoeboid migration to efficiently navigate through extracellular matrices and accumulate at inflammatory microenvironments.

This intrinsic ability to sense inflammatory signals and actively migrate toward diseased tissues provides the biological foundation for neutrophil‐based therapeutic strategies. In particular, the high expression of chemokine receptors, such as CXCR2, enables neutrophils to respond rapidly to gradients of inflammatory chemokines, including CXCL1 and CXCL8, which are frequently elevated in tumors, infections, and tissue injury [25]. Moreover, their capacity to penetrate biological barriers, including vascular and extracellular matrix barriers, allows neutrophils to access pathological regions that are difficult to reach through conventional drug delivery approaches.

However, excessive or dysregulated neutrophil recruitment can contribute to tissue damage and chronic inflammation. Neutrophil trafficking is tightly regulated by chemokines, inflammatory mediators, and adhesion molecules, which coordinate their directional migration, endothelial adhesion, and extravasation toward sites of infection, inflammation, and tissue injury. Understanding these mechanisms is essential for elucidating the context‐dependent functions of neutrophils and developing strategies to precisely regulate their recruitment and activity.

##### 2.2.1.2 Pathogen Recognition and Engulfment

Phagocytosis is a fundamental effector function of neutrophils that links pathogen recognition to intracellular elimination. Neutrophils recognize and internalize pathogens, cellular debris, and particulate materials through a range of surface receptors, including PRRs, Fcγ receptors, and complement receptors [13, 22, 26, 27, 28]. Receptor engagement triggers cytoskeletal remodeling, membrane extension, and phagosome formation, enabling the rapid sequestration of extracellular targets. Following internalization, phagosomes undergo rapid maturation and interact with neutrophil granules, generating a specialized antimicrobial compartment enriched in ROS and proteolytic enzymes. Unlike macrophage phagosomes, which undergo progressive acidification, neutrophil phagosomes maintain a relatively neutral to alkaline environment during the early stages of maturation, partly due to NADPH oxidase 2 (NOX2)‐mediated respiratory burst activity [29, 30]. The resulting ROS, together with granule‐derived antimicrobial enzymes such as neutrophil elastase (NE) and cathepsin G (CG), contributes to efficient intracellular pathogen killing [25, 31, 32].

Phagocytosis is closely interconnected with other neutrophil effector functions, including degranulation, oxidative responses, and extracellular trap formation, allowing neutrophils to coordinate intracellular and extracellular antimicrobial defense [33, 34, 35]. These processes also influence neutrophil activation, tissue responses, and the fate of internalized materials. Understanding the mechanisms underlying pathogen recognition, engulfment, and intracellular processing therefore provides an important foundation for elucidating neutrophil functions in health and disease and for developing therapeutic strategies that modulate or harness these intrinsic properties.

##### 2.2.1.3 Respiratory Burst‐Mediated Oxidative Killing

The respiratory burst is a hallmark effector function of activated neutrophils and plays a central role in oxidative antimicrobial defense. Upon activation, the NOX2 complex generates large amounts of ROS, including superoxide and hydrogen peroxide, which further contribute to the formation of potent oxidants such as hypochlorous acid (HOCl) through myeloperoxidase (MPO)‐mediated reactions [30, 36, 37, 38, 39]. Together with granule‐derived antimicrobial enzymes, these reactive species establish a highly oxidative intracellular and extracellular environment that promotes pathogen killing. ROS production is tightly regulated by the assembly and stability of the NOX2 complex. Multiple membrane and cytosolic components are required for NOX2 activation, while EROS (CYBC1) contributes to the stability and functional activity of the complex [40]. The magnitude of oxidative responses varies with neutrophil activation status and pathological context, reflecting differences in intracellular redox regulation and metabolic state [25, 41, 42]. Metabolic pathways are closely coupled to respiratory burst activity, as NADPH required for ROS generation is primarily supplied by the oxidative branch of the pentose phosphate pathway [43, 44]. Although mature neutrophils rely predominantly on glycolysis, metabolic‐sensing pathways such as the AMPK/SIRT1/PGC‐1α axis may further regulate energy homeostasis, stress responses, and functional plasticity under inflammatory conditions [45, 46, 47]. Beyond antimicrobial defense, excessive ROS production can contribute to tissue injury and influence neutrophil activation and inflammatory responses. Emerging evidence also suggests potential interactions between neutrophil redox homeostasis and regulated cell death pathways, including ferroptosis, although ferroptosis is not considered a predominant mode of neutrophil death [48, 49]. Thus, the respiratory burst represents an important link between neutrophil antimicrobial activity, metabolism, and inflammatory function. Its strong dependence on activation and local redox conditions may also provide a biological basis for developing ROS‐responsive therapeutic strategies in neutrophil‐rich pathological environments [41, 50].

#### 2.2.2 Nonclassical Extracellular Strategy: NETs

Beyond phagocytosis and degranulation, neutrophils can form NETs as a distinct extracellular defense mechanism (Figure 2B). First described by Brinkmann and colleagues in 2004, NETs are web‐like structures composed primarily of extracellular DNA associated with antimicrobial proteins, including NE, MPO, and CG [42, 51]. By immobilizing and concentrating antimicrobial components around extracellular targets, NETs extend neutrophil defense beyond intracellular pathogen elimination. This mechanism is particularly important for controlling large or aggregated pathogens, such as fungal hyphae and bacterial clusters, that are difficult to efficiently eliminate through conventional phagocytosis [51].

##### 2.2.2.1 Mechanisms of NETosis

NET formation involves coordinated changes in neutrophil signaling, granule components, chromatin organization, and membrane integrity. In the canonical suicidal pathway, neutrophil activation induces intracellular ROS production, with signaling pathways such as Raf–MEK–ERK contributing to NET formation [52, 53]. ROS‐dependent signaling promotes the redistribution of NE and MPO from granules toward the nucleus, where they contribute to chromatin remodeling [54]. Meanwhile, peptidylarginine deiminase 4 (PAD4) promotes histone citrullination, particularly citrullination of histone H3, which reduces histone‐DNA interactions and facilitates chromatin decondensation [55]. Subsequent disruption of the nuclear and plasma membranes allows decondensed chromatin and associated antimicrobial proteins to be released extracellularly, resulting in NET formation and, in the suicidal pathway, loss of neutrophil viability [56].

NET formation can also occur through nonlytic or “vital” pathways, in which neutrophils release extracellular chromatin while retaining viability and certain functions, including chemotaxis and phagocytosis [57]. The relative contribution of suicidal and vital NET formation depends on the activating stimulus and cellular context. These distinct modes highlight the functional plasticity of neutrophils and indicate that NET formation is not simply a terminal cell death process but a dynamic component of neutrophil responses.

##### 2.2.2.2 Biological Significance in Pathogen Entrapment

The biological effects of NETs are highly context dependent. Appropriate and transient NET formation contributes to pathogen immobilization and clearance, thereby complementing phagocytosis and degranulation [58, 59, 60, 61, 62]. However, excessive or persistent NET accumulation can transform this protective response into a source of tissue injury and chronic inflammation. NET‐associated DNA, histones, proteases, and other inflammatory components can promote endothelial damage, platelet activation, coagulation, and inflammatory signaling, thereby contributing to thrombosis and autoimmune or inflammatory diseases [59, 63, 64, 65, 66].

NETs may also interact with innate immune sensing pathways. NET‐derived DNA can activate the cGAS–STING pathway and amplify inflammatory responses, while interactions between NETs and macrophages or endothelial cells may further promote inflammatory polarization and vascular dysfunction [67, 68, 69]. In cancer, persistent NET formation has been associated with tumor cell adhesion, vascular dissemination, and metastatic colonization [59, 63, 64, 65, 66]. Thus, the effects of NETs depend not only on their formation but also on their magnitude, duration, composition, and surrounding tissue environment.

These findings underscore the dual role of NETs in host defense and disease progression. Therapeutic strategies should therefore aim to restrain excessive or dysregulated NET formation while preserving its physiological antimicrobial functions. The extracellular localization and distinctive biochemical composition of NETs may also provide opportunities for developing therapeutic approaches that selectively modulate NET‐associated responses.

#### 2.2.3 Immunomodulatory Functions

Transcriptomics and proteomics show that neutrophils produce chemokines and cytokines that interact with other leukocytes [2]. As part of the immune communication network, they signal to nearly all other immune cells. As first responders to infected or injured tissues, neutrophils establish conditions for inflammation and subsequent adaptive immunity [70]. This is regulated by a rapid transcriptional burst upon activation. While circulating neutrophils have low basal transcriptional activity, danger signals trigger synthesis of signaling molecules [71]. Although per‐cell cytokine production is lower than in macrophages, their large numbers at inflammatory sites make their overall contribution to the cytokine pool highly significant [19].

##### 2.2.3.1 Cytokine and Chemokine Secretion

Beyond their classical antimicrobial functions, neutrophils actively regulate inflammatory microenvironments through cytokines, chemokines, lipids, granular contents, and cell–cell interactions, thereby influencing the recruitment and function of other immune cells [10, 72, 73, 74]. Among these mediators, interleukin (IL)‐8 (CXCL8) promotes neutrophil recruitment and accumulation at inflammatory sites, whereas IL‐1β and TNF‐α can amplify local inflammatory signaling and reshape the surrounding immune landscape [75, 76, 77]. These functions highlight neutrophils as both therapeutic targets and potential cellular mediators for immunomodulatory delivery. Importantly, the effects of neutrophil‐associated inflammatory mediators are highly context dependent. IL‐6 provides a representative example, as transient signaling can support tissue adaptation and repair, whereas sustained signaling may promote chronic inflammation and pathological remodeling. These divergent effects depend on factors such as cytokine concentration, exposure duration, cellular source, and downstream signaling pathways [78]. Therefore, therapeutic intervention should favor context‐specific modulation rather than indiscriminate blockade, with the aim of suppressing pathological inflammation while preserving physiological immune functions. This principle may also inform neutrophil‐based delivery strategies by enabling selective modulation of inflammatory signaling at disease sites.

##### 2.2.3.2 The Neutrophils and Macrophages Interactions

As key effector cells of the innate immune system, neutrophils and macrophages engage in dynamic crosstalk that critically orchestrates both the initiation and the resolution of immune responses. Neutrophils modulate macrophage polarization toward either pro‐ or anti‐inflammatory phenotypes, thereby coordinating the progression or resolution of inflammation. For instance, studies have demonstrated that during a cytokine storm, neutrophil‐derived extracellular vesicles are internalized by macrophages. This uptake elevates intracellular itaconate levels and subsequently suppresses the production of inflammatory cytokines, ultimately dampening the progression of the storm [79]. Furthermore, in hepatocellular carcinoma, levels of NETs are elevated and correlate positively with the infiltration of M2‐type macrophages in tumor tissue, suggesting that NETs drive macrophage polarization and promote the progression of liver cancer via inflammatory signaling networks [80]. The cGAS–STING signaling pathway is a cornerstone of the innate immune system, essential for host defense against pathogens and for the maintenance of cellular homeostasis [81]. Emerging evidence indicates that the cGAS–STING pathway serves not only as a bridge connecting DNA sensing to innate immune responses, but also as a central hub regulating macrophage fate and function. Its aberrant activation is widely implicated in the pathogenesis of various diseases. For instance, in liver injury models, oxidized DNA damage products released by ferroptotic hepatocytes can activate STING signaling in macrophages. This activation promotes macrophage polarization toward the M1 phenotype, thereby exacerbating inflammation and fibrosis. The upstream activation signals for the cGAS–STING pathway primarily derive from endogenous and exogenous DNA, among which NETs represent another potent activation trigger. Guo et al. discovered that in postmenopausal osteoporosis models, NETosis of bone marrow neutrophils is activated. The released NET DNA promotes macrophage M1 polarization and osteoclastogenesis via the cGAS–STING/AKT2 pathway, which consequently aggravates bone resorption [67]. Beyond its well‐established role in macrophage polarization, the cGAS–STING pathway is also intimately linked to the regulation of neutrophil death, particularly NETosis, through the sensing of cytosolic DNA, modulation of inflammatory responses, and remodeling of the tissue microenvironment.

##### 2.2.3.3 The Neutrophils and Dendritic Cells Interactions

Neutrophils regulate dendritic cell (DC) recruitment, maturation, and antigen‐presenting functions, thereby influencing the transition from innate to adaptive immunity [82]. Upon activation, neutrophils release chemokines such as CCL3, CCL4, CCL5, and CCL20 to promote DC accumulation at inflammatory sites and facilitate subsequent immune activation [83, 84, 85]. In addition, neutrophil‐derived granule proteins and NETs can modulate DC maturation by regulating the expression of antigen‐presenting and costimulatory molecules, thereby shaping T‐cell responses [86, 87]. Neutrophils can also transfer antigens or membrane components to DCs through processes such as phagocytosis of neutrophil‐derived materials and trogocytosis, enhancing DC‐mediated immune priming [88, 89]. However, excessive activation of the neutrophil–DC axis may contribute to autoimmune and inflammatory disorders, highlighting the importance of precisely controlling this interaction in therapeutic applications [90, 91, 92]. Interestingly, in specific pathological contexts such as systemic lupus erythematosus, NET‐derived MPO can serve as a nonclassical ligand for TREM2 on DCs, activating a DAP12/SYK/ERK signaling cascade that promotes NET internalization and subsequent cGAS/STING activation, thereby lowering the threshold for DC activation [93]. A critical component of this interaction is the mechanism of antigen transfer, which facilitates the relocation of pathogenic material to the adaptive immune system for subsequent presentation. DCs frequently acquire antigens by phagocytosing either apoptotic or infected neutrophils, a “Trojan Horse” strategy that delivers preprocessed antigens in a milieu enriched with maturation signals [94, 95]. This exchange is supplemented by trogocytosis, a contact‐dependent process characterized by the active extraction and internalization of neutrophil plasma membrane fragments by DCs [96]. Ultimately, neutrophils function as environmental gatekeepers that not only summon and educate DCs but also dictate the spatial and functional transition from nonspecific innate defense to antigen‐specific adaptive immunity.

##### 2.2.3.4 The Neutrophils and Adaptive Immunity Interactions

Although traditionally considered innate immune effectors, neutrophils also participate in the regulation of adaptive immunity by shaping lymphocyte activation and immune polarization [97]. Through the release of cytokines, chemokines, extracellular vesicles, and other immune mediators, neutrophils influence the survival and functional responses of T and B cells within inflammatory microenvironments [98]. Under specific pathological conditions, activated neutrophils can acquire antigen‐presenting characteristics through the induction of MHC Class II and costimulatory molecules, enabling limited regulation of T‐cell responses [99]. In addition, neutrophil‐derived factors, including BAFF, APRIL, IL‐12, and NET‐associated mediators, contribute to adaptive immune activation and antibody response [82, 83]. These interactions highlight the functional plasticity of neutrophils and their ability to bridge innate and adaptive immunity. Understanding this immunoregulatory capacity provides opportunities for developing neutrophil‐targeted immunotherapies and delivery strategies that modulate immune responses within pathological site [86, 100].

##### 2.2.3.5 The Neutrophils and Platelets Interactions

Neutrophils and platelets form a functional communication network that regulates inflammation, thrombosis, and tumor progression. Platelet activation promotes neutrophil recruitment and activation through adhesion molecules and soluble mediators, facilitating NET formation and amplifying local inflammatory responses [87, 89, 101]. NETs further reinforce platelet activation and coagulation by providing a scaffold for cellular interactions, creating a positive feedback loop that contributes to pathological inflammation and thrombosis [91].

In the tumor microenvironment, platelet–neutrophil interactions promote tumor progression by enhancing NET formation, supporting tumor cell survival, and establishing an immunosuppressive niche [52, 102]. NETs can facilitate tumor cell dissemination and immune evasion by interacting with circulating tumor cells and impairing antitumor immune surveillance [103, 104, 105, 106]. Therefore, targeting the neutrophil–platelet–NET axis represents a potential strategy for controlling inflammation‐associated diseases and improving therapeutic efficacy. For example, glioma cells secrete high levels of IL‐8, which interacts with CXCR1/2 receptors to recruit neutrophils to the TME and trigger NETosis [102]. These tumor‐induced NETs subsequently promote glioma cell proliferation and maintain an immunosuppressive landscape [91]. This multicomponent interaction not only facilitates tumor‐associated thrombosis but also enhances metastasis by physically entrapping CTCs and increasing their adhesion to distant vascular beds. Crucially, the neutrophil–platelet axis plays a definitive role in regulating cancer dormancy and recurrence. Chronic inflammation, such as that induced by tobacco smoke or LPS exposure, promotes sustained NET formation in the lungs [52]. Mechanistic studies have revealed that NET‐associated proteases, specifically NE and MMP9, sequentially cleave the extracellular matrix component laminin‐111 [103]. This proteolytic remodeling exposes a cryptic epitope that activates α3∖β1 integrin signaling in quiescent cancer cells. This interaction subsequently triggers the FAK–ERK–MLC2–YAP signaling cascade, facilitating the reinitiation of proliferation and the transition from dormancy to active growth [104, 105].

#### 2.2.4 Resolution: Apoptosis and Efferocytosis

Resolution of inflammation is an active process that restores tissue homeostasis through the controlled removal of activated immune cells, particularly neutrophils, by efferocytosis [107]. Neutrophils normally undergo spontaneous apoptosis after completing their antimicrobial functions, preventing uncontrolled release of cytotoxic components such as ROS, proteases, and NETs [108, 109, 110]. During apoptosis, neutrophils expose “eat‐me” signals, mainly phosphatidylserine, which are recognized by macrophages and other efferocytes to promote efficient clearance and suppress excessive inflammation [111]. This process not only eliminates potentially harmful cellular debris but also induces anti‐inflammatory and proresolving programs in phagocytes through mechanisms involving MerTK and related signaling pathways [112, 113]. The balance between neutrophil activation, death, and clearance determines the duration and outcome of inflammatory responses. Therefore, manipulating neutrophil survival and clearance provides an important strategy for regulating inflammatory diseases and optimizing neutrophil‐based therapeutic delivery systems [114, 115, 116].

In the context of tissue homeostasis, the efferocytosis of apoptotic neutrophils by macrophages represents the definitive “switch” from inflammation to repair. The activation of cGAS–STING in macrophages significantly influences their polarization state, which in turn directly determines the mode of clearance for apoptotic neutrophils, a process known as efferocytosis, as well as the subsequent inflammatory microenvironment. In the context of physiological inflammatory resolution, macrophage‐mediated efferocytosis of apoptotic neutrophils relies on the recognition of exposed “eat‐me” signals like phosphatidylserine by receptors such as MerTK. Beyond clearing hazardous cargo like ROS, proteases, and NETs, this internalization orchestrates tissue remodeling and homeostatic recovery by steering macrophages toward a reparative M2 phenotype via immunometabolic reprogramming. Conversely, aberrant cGAS–STING pathway activation, fueled either by NETosis‐derived DNA or self‐DNA from secondary necrosis out of failed efferocytosis, functions as a potent trigger for the macrophage cGAS–STING axis. This sustained signaling forces an M1 polarization state characterized by MerTK suppression, compromised efferocytosis, and robust cytokine release. The cGAS–STING pathway thus operates as a pivotal molecular hub integrating neutrophil death inputs with macrophage functional outcomes, meaning that while tightly regulated activation preserves host defense, chronic hyperactivation flips the pathological switch toward nonresolving inflammation [117, 118]. In atherosclerosis, defective efferocytosis leads to the accumulation of apoptotic foam cells, which undergo secondary necrosis to form the necrotic cores characteristic of unstable plaques; recent evidence suggests that the proteolytic shedding of MerTK in the plaque microenvironment is a primary driver of this clearance failure. Similarly, in sepsis and acute lung injury (ALI), delayed neutrophil apoptosis or impaired efferocytosis prolongs the presence of destructive effector cells, leading to multiple organ failure and death [112].

## 3 The Pathophysiological Roles of Neutrophils in Human Diseases

Neutrophils contribute to the pathogenesis and progression of a broad spectrum of human diseases through context‐dependent changes in recruitment, activation, effector functions, and metabolic adaptation. This section first examines the roles of neutrophils across major disease settings, including solid tumors, cardiovascular and neurological disorders, chronic inflammatory and autoimmune diseases, and metabolic disorders and wound healing, highlighting how neutrophil responses can exert either protective or pathological effects depending on the disease stage and tissue microenvironment. We then summarize several common regulatory pathways shared across these diseases, including chemokine‐mediated recruitment, NET formation, protease activity, oxidative stress, and metabolic reprogramming. This organization moves from disease‐specific manifestations to convergent molecular mechanisms, providing a framework for understanding both the functional diversity of neutrophils and the common pathways that may serve as therapeutic targets across different pathological conditions.

### 3.1 Solid Tumors

A broad spectrum of malignancies is characterized by robust neutrophilic infiltration, a phenomenon so prevalent that these cells are formally recognized as tumor‐associated neutrophils (TANs). Clinically, a high density of intratumoral neutrophils has emerged as one of the most significant predictors of poor prognosis across diverse human cancers, with elevated neutrophil‐to‐lymphocyte ratios (NLR) consistently correlating with diminished overall survival [119]. The involvement of neutrophils begins at the very onset of malignant transformation, where the amplification of inflammatory signals and tissue stress serves as a primary driver of tumorigenesis. In experimental models of lung chemical carcinogenesis, neutrophils ROS that exacerbate DNA damage in pulmonary cells, directly catalyzing malignant conversion. In the context of RAS‐driven malignancies, neutrophils facilitate tumor cell proliferation by secreting various cytokines and growth‐promoting factors, including prostaglandin E2 (PGE2) and NE [120]. Furthermore, neutrophils retain significant antitumor potential, particularly during the early phases of cancer development. ROS produced by neutrophils, while potentially mutagenic, can also induce lethal DNA damage in nascent cancer cells [121]. NE has demonstrated selective cytotoxicity against cancer cells in both human and murine models while simultaneously priming CD8^+^ T cell‐mediated antitumor activity [122]. Moreover, neutrophils serve as critical regulators of the adaptive immune response; in lung cancer models, they have been shown to stimulate T cell proliferation and IFN‐γ production [123]. This defensive activity is frequently associated with an antigen‐presenting phenotype; indeed, early‐stage human lung cancers often harbor a distinct subset of “hybrid” neutrophils expressing HLA‐DR and CD74, which are capable of directly presenting tumor antigens to T cells [124]. Ultimately, neutrophil functions during cancer progression are shaped by clinical stage, tumor‐intrinsic signals, and the metabolic and immune landscape of the TME. Rather than representing fixed N1‐like or N2‐like states, neutrophils undergo context‐dependent functional changes in response to local and systemic cues. Several regulatory pathways involved in these responses, including chemokine‐mediated recruitment, ROS production, NE activity, and metabolic reprogramming, are also shared with other disease settings but may lead to distinct pathological outcomes [74].

### 3.2 Cardiovascular and Neurological Disorders

In the cardiovascular system, neutrophils act as multifaceted orchestrators of sterile inflammation, transitioning from the insidious progression of atherosclerosis to the hyperacute phases of myocardial injury. Atherosclerosis is driven by the intimal accumulation of lipids and metabolites, where neutrophils serve as early responders by adhering to activated endothelium via circadian‐regulated recruitment patterns [125]. During the early stages, neutrophil recruitment is notably governed by a neuro‐immune axis where sleep fragmentation reduces hypothalamic hypocretin, thereby releasing the inhibition on CSF1‐producing preneutrophils in the bone marrow and accelerating leukopoiesis. Within the lesion, the deposition of CG and neutrophil–platelet interactions involving CCL5 recruitment enhance vascular permeability and secondary monocyte influx. In advanced stages, activated smooth muscle cells (SMCs) recruit neutrophils that eject NETs containing histone H4; this externalized histone binds to and lyses SMCs, directly inducing plaque instability and rupture [126].

Neutrophils play an important role in myocardial ischemia–reperfusion (I/R) injury. Rather than being driven by a single cell death pathway, myocardial I/R injury (MIRI) involves interconnected processes including apoptosis, necroptosis, pyroptosis, and ferroptosis, which are regulated by shared signals such as oxidative stress, mitochondrial dysfunction, and inflammatory activation. Excessive ROS generation during reperfusion can promote lipid peroxidation and ferroptotic responses while amplifying inflammatory cell death and tissue injury. Neutrophil‐derived ROS, MPO, and inflammatory mediators may therefore contribute to this interconnected cell‐death network and exacerbate cardiomyocyte injury and postinfarction inflammation [18, 127]. This interplay highlights neutrophils as potential targets for interventions aimed at simultaneously modulating oxidative stress, inflammatory signaling, and cell‐death responses after myocardial reperfusion.

Parallel to their role in the vasculature, neutrophils exert a decisive influence on the central nervous system (CNS). In ischemic stroke (IS), which shares a thrombo‐inflammatory etiology with MI, neutrophils are the first leukocytes to breach the BBB. Vascular neutrophils interact with platelets to exacerbate microvascular obstruction, while infiltrated cells release elastase to degrade tight junction proteins, leading to cerebral edema [128]. NETosis within cerebral vessels further consolidates immunothrombi, conferring resistance to thrombolysis. In the chronic milieu of Alzheimer's disease (AD), neutrophils are recruited to β‐amyloid plaques via LFA‐1/ICAM‐1 interactions and the activation of complement components, including C5a and C1q; at these sites, persistent NETosis exacerbates neuroinflammation and synaptic loss [129].

Across cardiovascular and neurological disorders, neutrophil recruitment, NET formation, protease release, and oxidative stress represent convergent mechanisms of tissue injury. However, their downstream consequences are disease‐specific, ranging from thrombosis and plaque instability in cardiovascular disease to BBB disruption, microvascular obstruction, and neuroinflammation in neurological disorders [130].

### 3.3 Chronic Inflammation and Autoimmune Diseases

The transition of neutrophils from antimicrobial sentinels to mediators of tissue damage is prominent in chronic inflammatory and autoimmune diseases. In these disorders, dysregulated neutrophil activation and clearance, particularly aberrant NET formation, contribute to organ damage and autoantigen exposure. SLE exemplifies this pathology, characterized by impaired NET clearance and excessive NET formation [131]. In SLE patients, a specific subset of low‐density neutrophils or low‐density granulocytes (LDGs) exhibits an enhanced capacity for NETosis, ejecting oxidized mitochondrial DNA and antimicrobial peptides that form potent interferon‐stimulating complexes [132]. These complexes drive the chronic activation of plasmacytoid DCs and maintain a self‐perpetuating loop of IFN‐mediated autoimmunity. Furthermore, NET‐associated MMP‐9 induces endothelial cell apoptosis, while markers such as NGAL and double‐stranded DNA serve as critical predictors of lupus nephritis and systemic flares [133]. Thus, in SLE, excessive NET formation and impaired clearance link neutrophil activation to persistent autoantigen exposure and Type I interferon signaling. In RA, neutrophils infiltrate the synovium and contribute to cartilage destruction and loss of immune tolerance. NET‐associated citrullinated proteins promote the production of anticitrullinated protein antibodies [134], while the release of MPO, MMP‐9, and reactive nitrogen species contributes to chondrocyte and extracellular matrix damage. Together, these findings identify PAD4‐dependent NET formation as a shared mechanism linking neutrophil activation to loss of immune tolerance, although the downstream consequences differ across autoimmune diseases. This “double‐edged” nature of NETs extends to inflammatory bowel disease (IBD) and multiple sclerosis. In IBD, including ulcerative colitis (UC) and Crohn's disease (CD), CD177^+^ and CD62L^LO^CXCR4^HI^ neutrophil subsets respond to gut dysbiosis by releasing excessive ROS and bactericidal proteins [135]. These NETs disrupt the epithelial barrier, leading to intestinal hemorrhage and microvascular thrombosis a process that can be reversed by DNase I treatment [136]. Collectively, these findings indicate that NET formation, protease release, and oxidative stress are shared neutrophil mechanisms across chronic inflammatory and autoimmune diseases, whereas their pathological consequences depend on the tissue and disease context [137].

### 3.4 Metabolism and Wound Healing

Wound healing requires rapid metabolic adaptation to support neutrophil recruitment and effector functions. Injury‐associated signals, including DAMPs, H_2_O_2_, lipid mediators, and chemokines such as CXCL8, promote neutrophil recruitment, while the hypoxic wound environment favors glycolytic metabolism [138]. These metabolic adaptations support phagocytosis, respiratory burst, and NET formation. However, metabolic disorders such as metabolic syndrome and hyperglycemia can disrupt this balance, resulting in excessive neutrophil activation, tissue accumulation, and chronic inflammation. Importantly, chronic inflammation in metabolic disorders is not entirely detrimental, as inflammatory activation initially supports tissue defense, damage clearance, and restoration of homeostasis. However, persistent metabolic stress can transform this protective response into sustained immune activation and impaired tissue repair. Similar to macrophages, neutrophil responses are highly dependent on disease stage and microenvironmental cues. Therefore, therapeutic strategies should aim to restore the balance between protective and pathological inflammation rather than completely suppress neutrophil activity [139]. Hyperglycemia further induces glucotoxicity, diverting glucose into the polyol and hexosamine pathways, which alters neutrophil metabolism and promotes excessive, spontaneous, and structurally unstable NETosis [140]. Excessive oxidative stress is an important link between neutrophil activation and chronic tissue injury. Although ROS production is essential for antimicrobial defense, uncontrolled ROS accumulation can disrupt cellular homeostasis, amplify inflammatory signaling, and promote tissue damage. Thus, therapeutic strategies should aim to restore redox balance rather than completely suppress ROS production, thereby preserving host defense functions [139]. Persistent metabolic and oxidative stress may also disrupt endoplasmic reticulum (ER) homeostasis and activate the unfolded protein response (UPR). While transient ER stress can support cellular adaptation, sustained activation may promote metabolic dysfunction, inflammatory signaling, and cellular damage. In neutrophils, impaired ER homeostasis may affect maturation, effector functions, and inflammatory resolution, potentially contributing to chronic disease progression. Therefore, modulating ER stress to restore proteostasis and neutrophil function, rather than broadly inhibiting the UPR, may represent a more selective therapeutic approach [141].

### 3.5 Common Neutrophil Regulatory Pathways Across Diseases

Neutrophil‐mediated pathology across different diseases converges on several common regulatory pathways. CXCR1/2‐dependent recruitment controls neutrophil accumulation at sites of inflammation, whereas PAD4‐dependent NET formation, NE‐mediated proteolysis, and NOX2‐derived ROS contribute to tissue injury, thrombosis, inflammation, and immune regulation [130]. Metabolic pathways such as FATP2‐mediated lipid metabolism further shape neutrophil functional states, particularly in tumors and metabolic disorders (Table 1). These pathways therefore represent shared molecular nodes linking neutrophil responses across seemingly distinct diseases.

**TABLE 1:** Key neutrophil regulatory axes and their disease‐related functions.

		Disease‐related effects				
Regulatory axis	Primary function	Tumors	Cardiovascular	Neurological	Autoimmune	Metabolic
CXCR1/2	Recruitment [25]	Tumor infiltration [142]	Vascular inflammation [23]	Neuroinflammation	Leukocyte recruitment	Chronic inflammation [143]
PAD4/NETs	NET formation [55]	Metastasis [144]	Thrombosis [130]	BBB injury [145]	Autoantigen exposure [77]	Metabolic inflammation [146]
NE	Proteolysis [146]	Tumor invasion [147]	Tissue injury [18]	BBB disruption	Tissue damage	Adipose inflammation
NOX2/ROS	Oxidative burst [148]	TME remodeling [74]	Oxidative injury	Neural injury	Inflammatory damage	Metabolic stress [149]
FATP2	Lipid metabolism [3]	Immunosuppression [3]	—	—	Inflammatory regulation [150]	Metabolic dysfunction
Cytokine signaling	Activation/ reprogramming	TAN polarization [151]	Inflammation	Neuroinflammation	Chronic inflammation	Low‐grade inflammation [152]

Abbreviations: BBB, blood–brain barrier; IRI, ischemia–reperfusion injury; NE, neutrophil elastase; NETs, neutrophil extracellular traps; NOX2, NADPH oxidase 2; ROS, reactive oxygen species; TAN, tumor‐associated neutrophil; TME, tumor microenvironment.

Importantly, the pathological consequences of these pathways are highly dependent on disease and tissue context. For example, PAD4‐dependent NET formation contributes to metastasis in tumors, thrombosis in cardiovascular diseases, BBB injury in neurological disorders, and autoantigen exposure in autoimmune diseases [130]. Similarly, NE and ROS can promote tissue injury across multiple disorders but may also contribute to host defense or antitumor responses under specific conditions. Therefore, neutrophil‐associated pathways should not be considered uniformly pathogenic, and therapeutic strategies should aim to selectively modulate disease‐relevant neutrophil functions rather than broadly suppress neutrophil activity.

## 4 Broad Targeting: Strategies Targeting Neutrophils in Human Diseases

In recent years, neutrophil‐targeted interventions have expanded from experimental approaches to a diverse range of clinically evaluated therapies. However, clinical development has not followed a uniform trajectory, because the therapeutic value of neutrophil modulation depends strongly on disease context, neutrophil function, and the balance between host defense and tissue injury. As summarized in Figure 3A and Table 2, current strategies can be broadly classified into four categories: modulating neutrophil abundance, regulating recruitment and inflammatory signaling, neutralizing neutrophil‐derived mediators, and altering specific effector functions. These approaches range from established supportive therapies, such as G‐CSF and CXCR4 antagonists for neutropenia, to emerging strategies targeting CXCR1/2, C5aR1, NETs, neutrophil proteases, and TANs.

**FIGURE 3:**
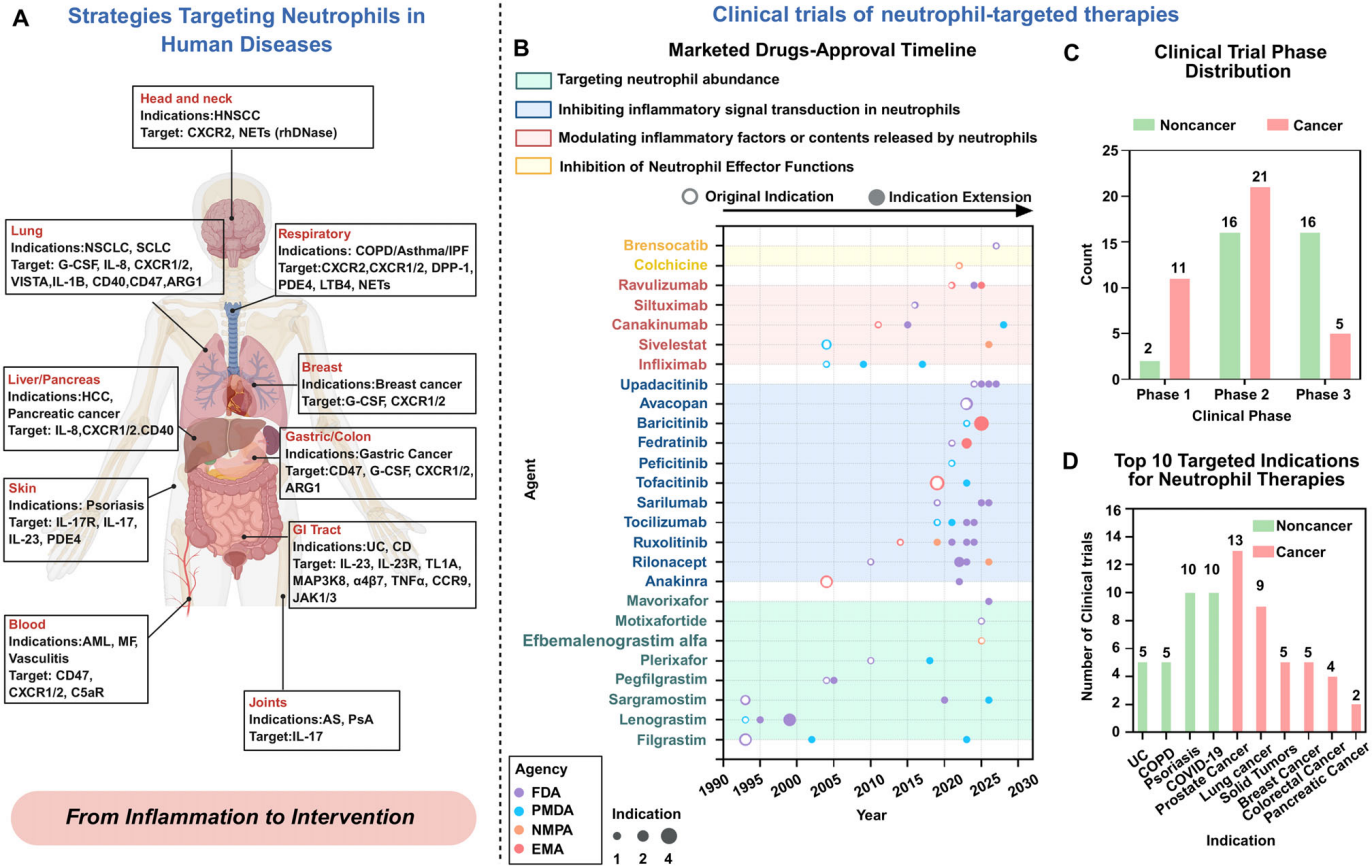
Therapeutic strategies targeting neutrophils and the clinical landscape. (A) Overview of neutrophil‐targeted strategies across human diseases, highlighting major indications and representative targets in different organs and systems. (B) Timeline of approved drugs targeting neutrophil‐related pathways, categorized by their primary mechanisms of action, including regulation of neutrophil abundance, inhibition of inflammatory signaling in neutrophils, modulation of neutrophil‐derived inflammatory mediators, and suppression of neutrophil effector functions. Dot colors indicate regulatory agencies and approval years, while dot size reflects the number of approved indications. Open circles denote original indications, and filled circles indicate indication extensions. (C) Distribution of clinical trials by phase, comparing cancer and noncancer indications. (D) Top disease indications for neutrophil‐targeted therapies ranked by the number of clinical trials.

**TABLE 2:** Approved drugs targeting neutrophil‐associated pathways.

Mechanism	Agent	Target	Agencies	Date of initial approval	Indications
Targeting neutrophil abundance	Efbemalenograstim alfa	CSF‐3 receptor agonist	NMPA	2023	Febrile neutropenia
	Filgrastim	CSF‐3 receptor agonist	US FDA	1991	Acute myeloid leukemia, bone marrow transplant, neutropenia
			PMDA	2000	Stem cell mobilization
			PMDA	2021	Neuroblastoma
	Lenograstim	CSF‐3 receptor agonist	PMDA	1991	Neutropenia
			PMDA	1993	Acute lymphocytic leukemia
			PMDA	1997	Urothelial carcinoma, Head and neck tumors, Acute myeloid leukemia
	Pegfilgrastim	CSF‐3 receptor agonist	US FDA	2002	Febrile neutropenia
			US FDA	2003	Stem cell mobilization
	Sargramostim	CSF‐2 receptor agonist	US FDA	1991	Hematopoietic stem cell transplantation, leukemia
			US FDA	2018	Acute radiation syndrome
			PMDA	2024	Acquired alveolar proteinosis
	Plerixafor	CXCR4 antagonist	US FDA	2008	Stem cell mobilization
			PMDA	2016	ASCT
	Motixafortide	CXCR4 antagonist	US FDA	2023	MM
	Mavorixafor	CXCR4 antagonist	US FDA	2024	WHIM syndrome
Inhibiting inflammatory signal transduction in neutrophils	Anakinra	Anti‐IL‐1R monoclonal antibody	EMA	2002	FMF, FCU, CAPS
			US FDA	2020	DIRA
	Rilonacept	IL‐1R trap fusion protein	US FDA	2008	CAPS
			US FDA	2020	FCU, DIRA
			US FDA	2021	Recurrent pericarditis
			NMPA	2024	MWS
	Tocilizumab	Anti‐IL‐6R monoclonal antibody	US FDA	2017	CRS
			US FDA	2022	COVID‐19
			PMDA	2017	TA
			PMDA	2019	AOSD
			US FDA	2021	SSc‐ILD
	Sarilumab	Anti‐IL‐6R monoclonal antibody	US FDA	2017	RA
			US FDA	2023	PMR
			US FDA	2024	PJIA
	Tofacitinib	JAK antagonist	EMA	2017	UC, PsA, PJIA, pauciarthritis
			NMPA	2021	AS
	Baricitinib	PMDA	2021	COVID‐19
			EMA	2023	PJIA, pauciarthritis, JIA, ERA
	Ruxolitinib		EMA	2012	cGVHD
			US FDA	2019	GVHD
			NMPA	2017	ET
			US FDA	2021	Atopic dermatitis
			US FDA	2022	Nonsegmental vitiligo
	Upadacitinib		US FDA	2022	Atopic dermatitis
			US FDA	2023	Crohn disease
			US FDA	2024	PJIA
			US FDA	2025	GCA
	Peficitinib		PMDA	2019	RA
	Fedratinib		US FDA	2019	Myelofibrosis
			EMA	2021	Splenomegaly, primary myelofibrosis,
	Avacopan	C5AR1 antagonist	US FDA	2021	microscopic polyangiitis, GPA, AAV
Modulating inflammatory factors or contents released by neutrophils	Canakinumab	Anti‐IL‐1β monoclonal antibody	EMA	2009	Gout
			US FDA	2013	SJIA
			PMDA	2026	Schnitzler syndrome
	Siltuximab	Anti‐IL‐6 monoclonal antibody	US FDA	2014	Multicentric Castleman disease
	Infliximab	Anti‐TNF‐α monoclonal antibody	PMDA	2002	Psoriasis
			PMDA	2007	Behcet disease
			PMDA	2015	MCLS
	Ravulizumab		EMA	2019	aHUS
			US FDA	2022	Myasthenia gravis
			EMA	2023	Neuromyelitis optica
	Sivelestat	Neutrophil elastase (NE) inhibitor	PMDA	2002	SIRS, AIL
			NMPA	2024	ARDS
Inhibition of neutrophil effector functions	Brensocatib	DPP‐1 inhibitor	US FDA	2025	Noncystic fibrosis bronchiectasis
	Colchicine	Microtubule polymerization inhibitor	NMPA	2020	Cardiovascular disease

Data sources: US FDA (https://www.fda.gov/); EMA (https://www.ema.europa.eu/); PMDA (https://www.pmda.go.jp/); NMPA (https://www.nmpa.gov.cn/).

Abbreviations: aHUS, atypical hemolytic uremic syndrome; ALI, acute lung injury; AOSD, adult‐onset still disease; ARDS, acute respiratory distress syndrome; ASCT, autologous stem cell transplant; CAPS, cryopyrin‐associated periodic syndromes; cGVHD, chronic graft versus host disease; CRS, cytokine release syndrome; CXCR4, C–X–C motif chemokine receptor 4; DIRA, deficiency of IL1 receptor antagonist; DPP‐1, dipeptidyl peptidase‐1; EMA, European Medicines Agency; ET, essential thrombocythemia; FCU, flexor carpi ulnaris; US FDA, United States Food and Drug Administration; FMF, familial Mediterranean fever; GM‐CSF, granulocyte–macrophage colony‐stimulating factor; HSC, hematopoietic stem cell; IBD, inflammatory bowel disease; MM, multiple myeloma; MWS, Muckle–Wells syndrome; NE, neutrophil elastase; NMPA, National Medical Products Administration (China); PJIA, polyarticular juvenile idiopathic arthritis; PMDA, Pharmaceuticals and Medical Devices Agency (Japan); PMR, polymyalgia rheumatica; RA, rheumatoid arthritis; SJIA, systemic juvenile idiopathic arthritis; TA, Takayasu arteritis.

Importantly, the clinical evidence supporting these approaches varies considerably. Therapies that restore insufficient neutrophil numbers have shown relatively clear clinical utility in defined neutropenic populations, whereas strategies that suppress neutrophil recruitment or activation have produced more heterogeneous outcomes. This difference reflects the dual role of neutrophils: excessive activity can cause tissue injury, but excessive suppression can compromise antimicrobial defense and tissue repair. Therefore, the clinical success of broad neutrophil modulation depends not only on target selection but also on disease stage, inflammatory burden, and patient phenotype. Figure 3B and Table 2 summarize representative clinically approved agents, while Figure 3C,D highlights emerging targets and major disease indications.

### 4.1 Anti‐Infective Therapy

Neutrophils are key effector cells of the innate immune system and represent the first line of defense against invading pathogens. They recognize pathogen associated molecular patterns through PRRs, triggering intracellular signaling and phagosome formation. Engulfed pathogens are eliminated via both oxygen dependent and oxygen independent mechanisms. However, some pathogens evade clearance by forming biofilms, releasing neutrophil targeted toxins, or using molecular mimicry to escape immune surveillance. Therapeutic strategies in infectious diseases mainly focus on two directions, enhancing neutrophil function and increasing neutrophil numbers. Conditions such as congenital neutropenia, severe infection, chemotherapy, and bone marrow suppression can markedly reduce neutrophil counts, highlighting the importance of restoring neutrophil levels in these settings.

Table 3 summarizes current clinical trials of neutrophil targeted therapies for noncancer conditions, including agents that promote neutrophil production, chemokine receptor inhibitors such as CXCR1 and CXCR2 antagonists, and complement pathway modulators, across infectious, inflammatory, and autoimmune diseases.

**TABLE 3:** Clinical trials of neutrophil‐targeted therapies for noncancerous conditions.

Target	Agents	Disease	Study objective	Phase	Status	NCT
α4β7	GS‐1427	Ulcerative colitis	Evaluate GS‐1427 efficacy in moderate‐to‐severe UC	Phase II	Active, not recruiting	NCT06290934
BTK	LOU064	Chronic spontaneous urticaria	Remibrutinib (LOU064) for antihistamine‐refractory CSU	Phase III	Completed	NCT05030311
			Remibrutinib (25 mg b.i.d.) for antihistamine‐refractory CSU	Phase III	Completed	NCT05032157
C5a	Ravulizumab	Thrombotic microangiopathy	Evaluate ravulizumab in HSCT‐TMA (efficacy/safety, PK/PD)	Phase III	Completed	NCT04543591
C5aR	CCX168	Vasculitis	Optimize remission induction therapy for non‐life‐threatening ANCA‐associated vasculitis	Phase II	Completed	NCT01363388
		ANCA‐associated vasculitis	Avacopan with standard therapy for remission in active AAV	Phase III	Completed	NCT02994927
		ANCA‐associated vasculitis	Evaluate avacopan long‐term safety in ANCA‐associated vasculitis (AAV)	Phase IV	Recruiting	NCT06072482
CCR9	AZD7798	Crohn's disease	AZD7798 for active ileal Crohn's (with ileostomy): safety, tolerability, mucosal repair	Phase II	Recruiting	NCT06681324
CXCR2	AZD5069	COPD	Evaluate AZD5069 safety and tolerability in COPD	Phase II	Completed	NCT01233232
	MK‐7123	COPD	Treatment outcomes	Phase II	Terminated	NCT01006616
	Danirixin	Pulmonary disease, chronic obstructive	Danirixin in high‐risk COPD: safety, PK/PD, efficacy	Phase II	Completed	NCT02130193
CXCR 1/2	Reparixin	PMF, ET‐MF, PV‐MF	Reparixin in high‐risk MF: efficacy, safety, tolerability	Phase II	Recruiting	NCT05835466
		Pneumonia, viral	Assess reparixin vs. placebo (plus standard care) in severe COVID‐19 pneumonia: efficacy and safety	Phase III	Completed	NCT04878055
CXCR4	Mavorixafor	Neutropenia	Evaluate mavorixafor for chronic neutropenia with recurrent infections	Phase III	Recruiting	NCT06056297
	Mavorixafor	WHIM syndrome	Mavorixafor efficacy in WHIM syndrome (neutrophil increase over placebo	Phase III	Active, not recruiting	NCT03995108
DPP‐1	XH‐S004	COPD	XH‐S004 in moderate‐to‐severe COPD: safety, PK/PD, preliminary efficacy	Phase I	Not yet recruiting	NCT07035652
IL‐6	Siltuximab	Cytokine release syndrome	Siltuximab for cytokine release syndrome (CRS)	Phase II	Active, not recruiting	NCT04975555
IL‐8						NCT02536469
IL‐17	Ixekizumab	Psoriasis	Ixekizumab onset in psoriasis via sequential photos	Phase III	Completed	NCT02387801
	Secukinumab	Ankylosing spondylitis	Secukinumab efficacy/safety in active AS (after NSAIDs/DMARDs/TNFα failure/intolerance)	Phase III	Completed	NCT01649375
		Psoriatic arthritis	Secukinumab PFS self‐administration in active PsA (24–52 weeks): efficacy, safety, tolerability	Phase III	Completed	NCT01752634
IL‐17R	AMG 827	Psoriasis	AMG 827 vs. placebo: % PASI improvement at Week 12	Phase II	Completed	NCT00975637
		Asthma	Determine AMG 827 vs. placebo: change in ACQ score	Phase II	Completed	NCT01199289
IL‐23	Mirikizumab	Ulcerative colitis	Tests mirikizumab in patients with high colon neutrophils	Phase IV	Completed	NCT06626165
	Guselkumab	Psoriasis	Guselkumab for moderate‐to‐severe plaque psoriasis: efficacy, safety, tolerability	Phase III	Completed	NCT02207231
		Ulcerative colitis	Evaluate guselkumab in moderately to severely active ulcerative colitis	Phase II/III	Active, not recruiting	NCT04033445
		Crohn's disease	Guselkumab in Crohn's disease: efficacy (GALAXI 1–3) and safety	Phase II/III	Active, not recruiting	NCT03466411
IL‐23R	Icotrokinra	Ulcerative colitis	Icotrokinra for moderate‐to‐severe ulcerative colitis (adults/adolescents): efficacy, safety, tolerability	Phase III	Recruiting	NCT07196748
IL‐1β	Canakinumab	Alcoholic hepatitis	Explore canakinumab in alcoholic hepatitis	Phase II	Completed	NCT03775109
JAK1/JAK3	Tofacitinib	Ulcerative colitis	Tofacitinib in Swedish UC patients: effectiveness and adherence	Observational	Recruiting	NCT04338204
LTB4	Zileuton	Asthma	Provide PK, safety/tolerability, and lung function activity data	Phase I/II	Completed	NCT00299065
		Pulmonary disease, chronic obstructive	Zileuton for reducing hospital stay in COPD exacerbation	Phase III	Terminated	NCT00493974
MAP3K8	Tilpisertib fosmecarbil	Ulcerative colitis	Evaluate tilpisertib fosmecarbil (GS‐5290) for moderate‐to‐severe UC: efficacy and safety	Phase II	Recruiting	NCT06029972
MPO	Mitiperstat	COPD	Evaluate mitiperstat efficacy/safety in COPD	Phase II	Completed	NCT05492877
			Evaluate AZD4831 efficacy/safety in HF with LVEF >40% (48 weeks)	Phase II/III	Completed	NCT04986202
NE	Sivelesta	Acute respiratory distress syndrome	Sivelestat sodium for ARDS with SIRS (multicenter RCT)	Phase IV	Recruiting	NCT04909697
		Cardiac disease	Assess sivelestat to prevent ALI/ARDS after cardiac surgery	Phase III	Completed	NCT06276569
	Brensocatib	Noncystic fibrosis bronchiectasis	Brensocatib for NCFB (52‐week, once‐daily)—ASPEN Study	Phase III	Completed	NCT04594369
NETs	rhDNase I	COVID‐19 infection	rhDNase1 in COVID‐19: safety, feasibility, NETs effect	Phase I	Completed	NCT04409925
			Treatment outcomes	Phase II	Unknown status	NCT04402944
			RhDNase for COVID‐19 respiratory failure: safety and 28‐day mortality	Phase II	Recruiting	NCT04445285
			Evaluate immune hyperactivation as COVID‐19 death mechanism	Phase III	Completed	NCT04402970
PDE3/PDE4	Ensifentrine	COPD	Treatment outcomes	Phase II	Recruiting	NCT05270525
			Ensifentrine for moderate‐severe COPD	Phase III	Completed	NCT04535986
			Ensifentrine safety/efficacy in moderate‐severe COPD	Phase III	Completed	NCT04542057
PDE4	Apremilast	Psoriasis	Evaluate apremilast (CC‐10004) for mild‐to‐moderate plaque psoriasis: efficacy and safety	Phase III	Completed	NCT03721172
	ARQ‐151	Chronic plaque psoriasis	ARQ‐151 cream vs. placebo once daily for 56 days in chronic plaque psoriasis	Phase III	Completed	NCT04211389
	Roflumilast	COPD	Roflumilast in COPD: exacerbation rate and lung function	Phase III	Completed	NCT00297115
	HSK44459	Idiopathic pulmonary fibrosis	Evaluate efficacy, with secondary objectives of safety and pharmacokinetics	Phase II	Not yet recruiting	NCT06764862
	Nerandomilast	Idiopathic pulmonary fibrosis	Assess long‐term tolerability of nerandomilast in pulmonary fibrosis	Phase III	Recruiting	NCT06238622
PI3Kδ	CDZ173	Common variable immunodeficiency (CVID), APDS / PASLI	CDZ173 (PI3Kδ inhibitor) for APDS/PASLI	Phase II/III	Completed	NCT02435173
TL1A	Duvakitug	Ulcerative colitis	Evaluate duvakitug in moderately to severely active ulcerative colitis	Phase III	Recruiting	NCT07184996
		Ulcerative colitis	evaluate the efficacy and safety of duvakitug in participants with moderately to severely active Ulcerative Colitis (UC)	Phase III	Recruiting	NCT07185009
	BCD‐261	Ulcerative colitis	BCD‐261 in moderate‐to‐severe UC (efficacy, safety, PK/PD, immunogenicity, dose‐response)	Phase II	Recruiting	NCT07080034
TNFα	SAR442970	Ulcerative colitis	Assess SAR442970 doses vs. placebo in moderate‐to‐severe UC	Phase II	Recruiting	NCT06975722

Data sources: ClinicalTrials.gov.

Abbreviations: AAV, ANCA‐associated vasculitis; ACQ, asthma control questionnaire; ALI, acute lung injury; ANCA, antineutrophil cytoplasmic antibodies; APDS, activated phosphoinositide 3‐kinase delta syndrome; ARDS, acute respiratory distress syndrome; AS, ankylosing spondylitis; BTK, Bruton's tyrosine kinase; C5a / C5aR, complement component 5a/5a receptor; COPD, chronic obstructive pulmonary disease; CRS, cytokine release syndrome; CSU, chronic spontaneous urticaria; DMARDs, disease‐modifying antirheumatic drugs; DPP‐1, dipeptidyl peptidase‐1; ET‐MF, essential thrombocythemia‐myelofibrosis; HF, heart failure; HSCT‐TMA, hematopoietic stem cell transplantation‐associated thrombotic microangiopathy; LVEF, left ventricular ejection fraction; MPO, myeloperoxidase; NCFB, noncystic fibrosis bronchiectasis; NE, neutrophil elastase; NETs, neutrophil extracellular traps; NSAIDs, nonsteroidal anti‐inflammatory drugs; PASI, psoriasis area and severity index; PDE, phosphodiesterase; PK/PD, pharmacokinetics/pharmacodynamics; PMF, primary myelofibrosis; PsA, psoriatic arthritis; PV‐MF, polycythemia vera‐myelofibrosis; rhDNase I, recombinant human deoxyribonuclease I; SCD, sickle cell disease; SIRS, systemic inflammatory response syndrome; UC, ulcerative colitis; WHIM, warts, hypogammaglobulinemia, infections, and myelokathexis syndrome.

#### 4.1.1 Enhance Neutrophil Activity

Therapeutic strategies aimed at enhancing neutrophil activity are primarily considered in patients with impaired innate immune defense, particularly those with neutropenia or defective antimicrobial responses. Rather than uniformly increasing neutrophil activation, current approaches seek to restore specific effector functions, including chemotaxis, phagocytosis, oxidative killing, and NET formation. This distinction is clinically important because excessive neutrophil activation can aggravate tissue injury, particularly in severe sepsis and COVID‐19. Thus, the therapeutic window for neutrophil activation is strongly dependent on disease stage and inflammatory status. Several pharmacological agents have been reported to enhance individual neutrophil functions. Fosfomycin can increase ROS production and oxidative burst, whereas erythromycin and several natural compounds, including gingerol, arecoline, and magnoflorine, have been reported to enhance phagocytic activity [153, 154]. Some antibiotics, including amoxicillin, clarithromycin, and enrofloxacin, can also promote NET formation [155, 156]. However, most of these observations remain mechanistic or preclinical rather than evidence of clinically established neutrophil‐directed therapy. Moreover, enhancing ROS production or NET formation may be beneficial for pathogen clearance but potentially harmful when neutrophil activation is already excessive. Therefore, these approaches should be interpreted as function‐modulating strategies rather than broadly applicable methods for neutrophil activation.

An alternative approach is to target pathological NET accumulation rather than suppressing neutrophils globally. Recombinant human deoxyribonuclease I (rhDNase I), which degrades extracellular DNA, has been evaluated in several clinical trials for severe COVID‐19. Aerosolized rhDNase I was investigated in nonintubated patients with respiratory distress (NCT04409925), while randomized studies evaluated its effects on pulmonary function and mechanical ventilation in critically ill patients (NCT04402944, NCT04402970) and on mortality in patients with COVID‐19‐related respiratory failure (NCT04445285) [157]. These studies illustrate an important principle for neutrophil‐targeted therapy: selective removal of a pathogenic neutrophil‐derived product may preserve essential antimicrobial functions while limiting tissue injury. Nevertheless, the clinical benefit of NET‐directed interventions may depend on disease stage, tissue localization, and the extent to which NET accumulation contributes to pathology.

#### 4.1.2 Increasing Neutrophil Abundance

Increasing neutrophil abundance remains the most clinically established strategy for patients with quantitative neutrophil deficiency. Recombinant G‐CSF therapies, including filgrastim and lenograstim, promote neutrophil production through CSF3R activation and have become standard supportive therapies for chemotherapy‐associated neutropenia. Long‐acting formulations, such as pegfilgrastim, lipegfilgrastim, and telpegfilgrastim, were subsequently developed to prolong systemic exposure and reduce administration frequency. In a Phase III trial (NCT04466137), YPEG–rhG–CSF demonstrated efficacy and safety in patients receiving chemotherapy and was approved in China in 2023 for the prevention of febrile neutropenia in patients with nonmyeloid malignancies. These agents provide a clear clinical benefit when neutropenia is the principal cause of impaired host defense, although their utility is less evident when neutrophil dysfunction results from pathological activation rather than insufficient cell numbers.

A complementary strategy is to mobilize neutrophils by disrupting bone marrow retention. The CXCR4/CXCL12 axis regulates neutrophil and hematopoietic cell retention within the bone marrow, and its inhibition can increase peripheral neutrophil availability. Plerixafor (AMD3100) is clinically established for hematopoietic stem cell mobilization and has also been evaluated in WHIM syndrome. In a Phase III trial (NCT02231879), prolonged CXCR4 inhibition improved neutropenia and reduced infection burden in patients with WHIM syndrome. More recently, motixafortide, a long‐acting CXCR4 antagonist, has expanded the clinical application of this approach by improving HSPC mobilization, while the orally available CXCR4 antagonist mavorixafor was approved in 2024 for WHIM syndrome and demonstrated sustained improvement in peripheral neutrophil counts. Ongoing trials are evaluating its potential in broader chronic neutropenia populations (NCT03995108, NCT06056297).

These approaches highlight an important distinction between increasing neutrophil numbers and correcting neutrophil function. G‐CSF and CXCR4 antagonists are particularly effective when quantitative deficiency limits host defense, but increasing circulating neutrophils may not resolve defects in chemotaxis, phagocytosis, or microbial killing. Conversely, excessive mobilization could theoretically increase inflammatory cell accumulation in susceptible tissues. Therefore, patient selection based on the underlying mechanism of neutrophil dysfunction is essential for maximizing therapeutic benefit while minimizing inflammatory complications. Emerging approaches aim to achieve more selective regulation of neutrophil production and maturation. For example, wedelolactone has been reported to promote neutrophil recovery after radiotherapy through TLR2‐dependent MEK/ERK signaling and activation of myeloid transcriptional programs [158]. However, evidence for such approaches remains predominantly preclinical. Further validation is required to determine whether pharmacological manipulation of neutrophil differentiation can provide advantages over established G‐CSF‐based therapies in terms of efficacy, durability, and safety.

Collectively, current clinical evidence indicates that increasing neutrophil abundance is most effective when neutropenia is a clearly defined therapeutic target, whereas functional enhancement or NET‐directed intervention requires greater consideration of disease stage and inflammatory context. These differences highlight a broader limitation of conventional neutrophil modulation: strategies that alter neutrophil numbers or shared effector pathways cannot fully account for their substantial functional heterogeneity. This limitation provides a rationale for developing more selective approaches capable of distinguishing pathogenic from protective neutrophil responses, as well as delivery systems that exploit neutrophil trafficking without globally altering their function.

### 4.2 Anti‐Inflammatory Therapy

Inflammation is essential for pathogen clearance and tissue repair, yet its chronic or excessive activation drives diseases ranging from autoimmune disorders to hematological malignancies. As key mediators, neutrophils drive inflammation by releasing cytokines and granular contents. Consequently, targeting neutrophil dysfunction has become a central strategy in anti‐inflammatory therapy. Current approaches focus on two primary pathways: inhibiting inflammatory signal transduction and modulating the release of inflammatory mediators. Their therapeutic value, however, varies substantially across disease settings, reflecting differences in neutrophil involvement, target specificity, and the balance between inflammatory injury and host defense.

#### 4.2.1 Drugs Inhibiting Signal Transduction of the Inflammatory Response in Neutrophils

Neutrophil activation is regulated by interconnected signaling pathways initiated by GPCRs, TLRs, and cytokine receptors and propagated through MAPK, NF‐κB, PI3K/Akt, and JAK–STAT signaling. Pharmacological intervention at these nodes can reduce pathological recruitment, degranulation, ROS production, or NET formation, but the clinical therapeutic window depends strongly on the relative contribution of neutrophils to disease pathology. Direct inhibition of neutrophil‐derived proteases represents one of the most clinically developed approaches. Sivelestat sodium, a selective NE inhibitor, is approved in China for ALI and acute respiratory distress syndrome (ARDS) associated with systemic inflammatory response syndrome (SIRS). Clinical studies have reported improvements in oxygenation and selected respiratory outcomes, although evidence for mortality benefit remains inconsistent [159]. These findings illustrate a major limitation of direct effector inhibition: suppression of tissue‐damaging protease activity may provide organ protection without necessarily modifying the upstream inflammatory process that drives disease progression. By contrast, brensocatib (Brinsupri), a reversible inhibitor of dipeptidyl peptidase‐1 (DPP1), acts upstream during neutrophil maturation and reduces activation of NE and other serine proteases. In the Phase III ASPEN trial (NCT04594369), brensocatib significantly reduced the annualized rate of pulmonary exacerbations in patients with noncystic fibrosis bronchiectasis (NCFB) [160]. Compared with direct inhibition of extracellular NE, upstream inhibition of protease maturation may provide more sustained control of neutrophil protease activity. However, its clinical benefit has been demonstrated primarily in bronchiectasis, emphasizing that target selection and disease context strongly influence therapeutic efficacy. The CXCR1/CXCR2 axis provides another strategy for limiting pathological neutrophil recruitment. Navarixin (MK‐7123), for example, reduced airway neutrophil accumulation in COPD but was associated with dose‐dependent reductions in circulating absolute neutrophil counts (ANCs), narrowing its therapeutic window [161]. This experience highlights the central challenge of systemic chemokine receptor blockade: the same pathways that mediate pathological tissue infiltration also contribute to protective neutrophil trafficking. Thus, reducing neutrophil recruitment may be beneficial in diseases dominated by excessive tissue infiltration but may become detrimental when systemic host defense is simultaneously compromised. The clinical development of CXCR1/CXCR2 inhibitors has consequently increasingly emphasized disease context, dosing, and combination strategies rather than indiscriminate neutrophil depletion.

Phosphodiesterase inhibition provides an example of indirect modulation of neutrophil‐associated inflammation. Ensifentrine, an inhaled dual PDE3/PDE4 inhibitor approved by the US FDA in 2024, increases intracellular cAMP and suppresses the release of proinflammatory mediators. Clinical trials in moderate‐to‐severe COPD (NCT05270525, NCT04535986, and NCT04542057) demonstrated improvements in lung function and reductions in exacerbations [162]. However, PDE3/4 signaling is not restricted to neutrophils, and the clinical benefits of ensifentrine arise from combined bronchodilatory and anti‐inflammatory effects. It should therefore be considered a neutrophil‐associated rather than neutrophil‐specific intervention. Intracellular kinase inhibition has similarly expanded the therapeutic landscape. Leniolisib, a selective PI3Kδ inhibitor, is approved for activated PI3Kδ syndrome and reduces pathological p110δ signaling (NCT02435173). JAK inhibitors such as ruxolitinib and baricitinib have also been used to control systemic hyperinflammation, including severe COVID‐19 and cytokine release syndromes, partly by suppressing cytokine‐driven JAK–STAT signaling and aberrant neutrophil activation [163]. However, their broad immunomodulatory activity means that therapeutic effects cannot be attributed solely to neutrophil regulation. Likewise, remibrutinib, a highly selective BTK inhibitor, illustrates the potential of targeting proximal inflammatory signaling in chronic inflammatory diseases, although its clinical activity primarily reflects broader immune modulation rather than neutrophil‐specific inhibition (NCT05030311 and NCT05032157) [164].

The C5a–C5aR1 axis represents a more closely linked neutrophil‐associated pathway. C5a is a potent neutrophil chemoattractant and activator, and its receptor C5aR1 has therefore emerged as an important therapeutic target. Avacopan, an oral C5aR1 antagonist, is approved as an adjunctive treatment for adults with severe ANCA‐associated vasculitis, including granulomatosis with polyangiitis and microscopic polyangiitis. By selectively blocking C5aR1 signaling, avacopan suppresses neutrophil activation while potentially preserving other complement functions. Its clinical development has also extended to complement‐mediated diseases including atypical hemolytic uremic syndrome, IgA nephropathy, and C3 glomerulopathy, with ongoing studies evaluating its broader therapeutic utility and long‐term safety (NCT02994927, NCT01363388, and NCT06072482). Although C5aR1 inhibition provides greater mechanistic proximity to neutrophil regulation than broad cytokine blockade, C5a signaling also affects other immune and vascular processes. Thus, its therapeutic effects should not be interpreted as exclusively neutrophil mediated. At the molecular effector level, PAD4 and MPO have emerged as promising targets. PAD4 inhibitors such as GSK484 and Cl‐amidine suppress histone citrullination and NET formation and have shown protective effects in preclinical models of sepsis and sterile inflammation [165]. However, these compounds have not yet established clinical efficacy, and excessive suppression of NET formation could potentially interfere with antimicrobial defense. Similarly, the MPO inhibitor mitiperstat (AZD4831) has been evaluated in COPD and cardiovascular diseases. The Phase IIa CRESCENDO study (NCT05492877) investigated mitiperstat in moderate‐to‐severe COPD and has been completed, providing an important clinical test of MPO inhibition in chronic pulmonary inflammation [166]. Natural products such as eupatilin have also been investigated for their ability to modulate NF‐κB and MAPK signaling, although their relatively broad mechanisms and limited disease‐specific clinical evidence remain important translational considerations [167].

Collectively, these studies indicate that the clinical utility of neutrophil‐associated anti‐inflammatory strategies depends on the level at which the inflammatory cascade is targeted. Direct effector inhibition may provide rapid tissue protection, upstream protease inhibition may offer more sustained control, and recruitment or signaling blockade may be more appropriate when excessive neutrophil accumulation or activation is a dominant pathogenic mechanism. However, strategies acting on shared inflammatory pathways generally have lower cellular specificity and may impair protective immune responses. Thus, disease stage, neutrophil contribution to pathology, and the balance between inflammatory injury and host defense should be considered when selecting the therapeutic target.

#### 4.2.2 Drugs Modulating Inflammatory Factors or Contents Released by Neutrophils

Beyond direct modulation of neutrophil signaling, several clinically established therapies target inflammatory mediators or effector pathways associated with neutrophil activation. These approaches are particularly relevant when neutrophils function as downstream effectors of broader inflammatory networks. Importantly, these interventions should be distinguished from neutrophil‐specific therapies because their targets are shared across multiple immune and tissue compartments.

Cytokine neutralization represents a clinically mature strategy. Canakinumab, a selective anti‐IL‐1β monoclonal antibody, is approved for several autoinflammatory disorders, including cryopyrin‐associated periodic syndromes and adult‐onset Still's disease, and has also been evaluated in alcoholic hepatitis (NCT03775109) [168]. Similarly, siltuximab, an anti‐IL‐6 antibody approved for idiopathic multicentric Castleman disease, has been investigated for CAR‐T cell‐associated cytokine release syndrome (NCT04975555) [168]. Although these agents can indirectly reduce neutrophil activation by suppressing upstream cytokine signaling, their effects extend beyond neutrophils and therefore represent broader inflammatory control rather than selective neutrophil modulation. Complement inhibition provides another clinically validated approach. Eculizumab and ravulizumab target C5 and are established treatments for complement‐mediated disorders, with ravulizumab also undergoing evaluation in hematopoietic stem cell transplant‐associated thrombotic microangiopathy (NCT04543591). These agents can indirectly affect neutrophil recruitment and activation through the C5a pathway, but their therapeutic effects reflect broader complement regulation. Direct inhibition of neutrophil‐derived effectors is more closely linked to neutrophil biology. Sivelestat has been used clinically to inhibit NE activity in SIRS‐associated ARDS, with recent clinical data further evaluating its potential to reduce postoperative ARDS and mortality [159, 169]. Brensocatib provides an alternative upstream approach by inhibiting DPP1‐dependent maturation of NE and other serine proteases. In the Phase III ASPEN trial (NCT04594369), brensocatib reduced exacerbation rates in patients with NCFB [160]. These findings suggest that controlling the production or maturation of neutrophil proteases may be more durable than inhibiting individual extracellular effectors, although the clinical benefit remains strongly disease dependent.

MPO and NET‐associated pathways represent additional emerging targets. MPO inhibitors such as AZD5904 and mitiperstat (AZD4831) have been investigated for reducing oxidative stress‐associated tissue injury in COPD and cardiovascular disease (NCT05492877) [166]. PAD4 inhibitors, including GSK484 and Cl‐amidine, have shown efficacy in preclinical models by limiting histone citrullination and NET formation [165], but clinical translation remains limited. The major challenge is to suppress pathological NET‐associated inflammation without compromising their physiological antimicrobial functions.

Overall, clinical experience indicates that therapies targeting neutrophil‐associated mediators may provide greater translational feasibility than approaches aimed at globally suppressing neutrophil activity, but their efficacy remains highly dependent on disease context. The emerging clinical paradigm therefore favors selective intervention at pathogenic effector pathways while preserving essential neutrophil functions. This consideration also highlights the potential value of local or targeted delivery approaches, which may improve the spatial selectivity of neutrophil‐associated therapies and reduce systemic exposure.

### 4.3 Antitumor Therapy

The tumor immune microenvironment (TIME) is a dynamic ecosystem in which neutrophils can exert either tumor‐promoting or tumor‐suppressive functions. Unlike infectious and autoimmune diseases, in which the therapeutic objective is generally to restore or restrain neutrophil activity, cancer therapy must account for substantial neutrophil heterogeneity and functional plasticity. Current strategies therefore focus on four major approaches: depletion of immunosuppressive neutrophil populations, blockade of neutrophil recruitment, inhibition of tumor‐supportive effector functions, and phenotypic reprogramming toward antitumor activity. Table 4 summarizes the current clinical progress of the aforementioned neutrophil‐targeted intervention strategies in cancer treatment.

**TABLE 4:** Clinical trials of neutrophil‐targeted therapies in cancer.

Target	Agents	Disease	Study objective	Phase	Status	NCT
ARG1	INCB001158	NSCLC, colorectal cancer (CRC)	Treatment outcomes	Phase I	Completed	NCT02903914
		Biliary tract cancer	Evaluate INCB001158 + chemo in advanced/metastatic solid tumors	Phase I/II	Completed	NCT03314935
CCR5	Maraviroc	Metastatic colorectal cancer	Treatment outcomes	Phase I	Completed	NCT03274804
CD40	APX005M	Metastatic pancreatic adenocarcinoma	Metastatic pancreatic cancer: drug combo efficacy	Phase Ib/II	Completed	NCT03214250
		Non‐small cell lung cancer	Safety and efficacy	Phase I/II	Completed	NCT03123783
	Selicrelumab	Solid tumors	Assess selicrelumab + atezolizumab in advanced solid tumors (safety/PK/PD/activity)	Phase I	Completed	NCT02304393
CD47	Magrolimab	mNSCLC	Magrolimab + docetaxel for solid tumors: safety, efficacy	Phase II	Terminated	NCT04827576
		Acute myeloid leukemia	Magrolimab + venetoclax + azacitidine vs. placebo in untreated AML (chemo unfit)	Phase III	Terminated	NCT05079230
	ALX148	Gastric cancer	Treatment outcomes	Phase II/III	Active, not recruiting	NCT05002127
	AO‐176	Non‐small cell lung adenocarcinoma	Evaluate safety, PK/PD, and clinical effects	Phase I/II	Completed	NCT03834948
	STI‐6643	Solid tumor	Treatment outcomes	Phase I	Unknown status	NCT04900519
CXCR1/2	SX‐682	Breast cancer	SX‐682 + M7824 + CV301 for advanced solid tumors	Phase I/II	Completed	NCT04574583
		Metastatic melanoma	Determine safety and efficacy of SX‐682 for melanoma	Phase I	Active, not recruiting	NCT03161431
		Non‐small cell lung cancer	Treatment outcomes	Phase II	Recruiting	NCT05570825
		Metastatic pancreatic ductal adenocarcinoma	SX‐682 + nivolumab MTD in metastatic PDAC (≥16 weeks chemo, no progression)	Phase I	Recruiting	NCT04477343
		Pancreatic cancer	Tislelizumab + SX‐682 for resectable pancreatic cancer	Phase II	Active, not recruiting	NCT05604560
		Metastatic colorectal cancer	SX‐682 ± nivolumab safety/MTD in RAS‐mutant MSS mCRC	Phase Ib/II	Active, not recruiting	NCT04599140
	Sotorasib	NSCLC	Phase I: determine RP2D of ladarixin + sotorasib. Phase II: evaluate PFS	Phase I/II	Recruiting	NCT05815173
	Paclitaxel + reparixin	Metastatic breast cancer	Reparixin + paclitaxel safety/PK in HER2‐metastatic breast cancer	Phase I	Completed	NCT02001974
CXCR2	MK‐7123	Metastatic solid tumors	Navarixin + pembrolizumab for three solid tumors	Phase II	Completed	NCT03473925
	AZD5069	HNSCC	Assess MEDI4736 + AZD9150 or AZD5069 (safety, PK, antitumor activity)	Phase I/II	Completed	NCT02499328
		Metastatic castration‐resistant prostate cancer	AZD5069 + enzalutamide: safety and optimal dose in mCRPC	Phase I/II	Terminated	NCT03177187
		Metastatic pancreatic ductal adenocarcinoma	MEDI4736 ± chemo/AZD5069 for PDAC: safety and activity	Phase I/II	Completed	NCT02583477
G‐CSF	Pegfilgrastim	Breast cancer	Pegfilgrastim for neutropenia prevention	Phase III	Completed	NCT00035594
	F‐627	Breast cancer	Evaluate efficacy and safety of F‐627	Phase III	Completed	NCT03252431
	Placebo	Colon cancer	Pegfilgrastim for severe neutropenia after 3 chemotherapies	Phase II	Completed	NCT00094809
	Filgrastim	Metastatic colorectal cancer	G‐CSF prevents neutropenia in first‐line metastatic CRC treatment	Phase II	Completed	NCT00541125
	YPEG–rhG–CSF	Non‐small cell lung cancer	YPEG–rhG–CSF and PEG–rhG–CSF for chemo‐induced neutropenia	Phase II	Completed	NCT02005458
	MK‐3475	Small cell lung cancer	To assess the safety and tolerability of monotherapy	Phase I	Completed	NCT01840579
IL‐8/CXCL8	BMS‐986253	Hepatocellular carcinoma	Nivolumab combinations and nivolumab alone for ORR in advanced HCC	Phase II	Terminated	NCT04050462
		Hormone‐sensitive prostate cancer	Assess nivolumab (±BMS‐986253) plus ADT for PSA‐rising hormone‐sensitive prostate cancer	Phase I/II	Active, not recruiting	NCT03689699
		Non‐small cell lung cancer	Preoperative nivolumab with CCR2/5 inhibitor or anti‐IL‐8	Phase II	Active, not recruiting	NCT04123379
	HuMax‐IL8	Solid tumor	Safety and efficacy of HuMax‐IL8	Phase I	Completed	NCT02536469
IL‐1β	Canakinumab	NSCLC	Neoadjuvant canakinumab ± pembrolizumab: MPR and TME changes	Phase II	Terminated	NCT03968419
		Lung cancer	Treatment outcomes	Phase III	Terminated	NCT05725343
LTB4	LY293111	NSCLC	LY293111 + gem/cis in NSCLC: efficacy and safety	Phase II	Completed	NCT00069875
NETs	rhDNase	Head and neck cancer	Dornase alfa vs. placebo for Stage III/IV head and neck chemoradiotherapy	Early Phase I	Unknown status	NCT00536952
TGF‐βR	TEW‐7197	Advanced stage solid tumors	TEW 7197 Phase I: safety, PK in advanced refractory solid tumors	Phase I	Completed	NCT02160106
	OT‐101	Malignant pleural mesothelioma	Efficacy assessment	Phase II	Not yet recruiting	NCT05425576
	MSB0011359C	Solid tumors	MSB0011359C: safety, PK, activity in advanced tumors + expansions	Phase I	Completed	NCT02517398
VISTA	HMBD‐002	TNBC/NSCLC	Studies on dosage and antitumor activity	Phase I	Completed	NCT05082610

Data sources: ClinicalTrials.gov.

Abbreviations: ADT, androgen deprivation therapy; AML, acute myeloid leukemia; ARG1, Arginase 1; CCR, C–C motif chemokine receptor; CCR2/5, C–C motif chemokine receptor 2 and 5; CRC, colorectal cancer; CXCL8, C–X–C motif chemokine ligand 8; CXCR, C–X–C motif chemokine receptor; G‐CSF, granulocyte colony‐stimulating factor; HCC, hepatocellular carcinoma; HER2, human epidermal growth factor receptor 2 negative; HNSCC, head and neck squamous cell carcinoma; IL‐1β / IL‐8, interleukin‐1 beta/interleukin‐8; LTB4, leukotriene B4; mCRC, metastatic colorectal cancer; mCRPC, metastatic castration‐resistant prostate cancer; mNSCLC, metastatic non‐small cell lung cancer; MPR, major pathological response; MSS, microsatellite stable; MTD, maximum tolerated dose; NETs, neutrophil extracellular traps; NSCLC, non‐small cell lung cancer; ORR, objective response rate; PDAC, pancreatic ductal adenocarcinoma; PFS, progression‐free survival; PK/PD, pharmacokinetics/pharmacodynamics; PSA, prostate‐specific antigen; rhG‐CSF, recombinant human granulocyte colony‐stimulating factor; RP2D, recommended Phase 2 dose; TGF‐βR, transforming growth factor‐beta receptor; TME, tumor microenvironment; TNBC, triple‐negative breast cancer; VISTA, V‐domain Ig suppressor of T cell activation.

#### 4.3.1 Blocking Neutrophil Infiltration Into Local Tumors

Selective reduction of TAN or PMN‐MDSC accumulation represents an important strategy for remodeling the immunosuppressive TIME. Conventional chemotherapeutics such as gemcitabine and 5‐fluorouracil can reduce immunosuppressive myeloid populations and thereby enhance antitumor immune responses. Gemcitabine induces apoptosis of PMN‐MDSCs through TP53INP1 and GSK‐3β signaling, whereas 5‐FU selectively reduces MDSCs and can enhance IFN‐γ production by tumor‐infiltrating T cells [170, 171]. However, these approaches are not neutrophil‐specific and may produce broader cytotoxic and immunomodulatory effects. Their therapeutic value therefore depends on the balance between depletion of immunosuppressive populations and preservation of beneficial immune cells.

The CXCL8/CXCR1/2 axis is a more direct approach to limiting neutrophil and PMN‐MDSC recruitment. CXCR1/2 inhibition with SX‐682 has shown promising preclinical activity in HNSCC by reducing PMN‐MDSC infiltration and enhancing the efficacy of adoptive NK‐ or T‐cell therapy [172]. However, clinical translation remains under investigation, and systemic blockade of CXCR1/2 may face the same challenge observed with other chemokine inhibitors: these receptors contribute to physiological neutrophil trafficking as well as tumor recruitment. Reparixin provides an instructive example of the importance of patient selection. In metastatic breast cancer, the Phase Ib study of reparixin combined with paclitaxel showed an initial response rate of approximately 30% [173]. However, the subsequent Phase II fRida trial (NCT02370238) failed to significantly improve progression‐free survival, potentially reflecting the low prevalence of the targeted ALDH‐positive cancer stem‐cell population in the enrolled cohort [174]. This discrepancy illustrates that inhibition of a neutrophil‐associated pathway may not translate into clinical benefit when the targeted biological population is insufficiently represented. Biomarker‐defined patient selection and confirmation of target engagement are therefore critical for future neutrophil‐directed oncology trials.

The CXCL12/CXCR4 axis provides another strategy for modifying the tumor–stromal interface. CXCR4 antagonists such as AMD3100 (plerixafor) and the high‐affinity peptide antagonist motixafortide have shown preclinical activity by disrupting protective interactions between tumor cells and myeloid cells in the bone marrow niche. In prostate and breast cancer models, these agents can enhance chemosensitivity, and AMD3100 combined with docetaxel has demonstrated greater antitumor activity than docetaxel alone [175]. However, because CXCR4 regulates multiple hematopoietic and stromal cell populations, its therapeutic effects cannot be considered neutrophil‐specific.

#### 4.3.2 Modulating Neutrophil Function for the Treatment of Cancer

Beyond recruitment blockade, functional modulation aims to interfere with neutrophil‐mediated immunosuppression while preserving or enhancing antitumor functions. PDE4 inhibition illustrates the challenges of translating neutrophil‐targeted mechanisms across species. Although roflumilast is clinically approved for COPD, its effects on human neutrophil chemotaxis, ROS production, and NE activity are less pronounced than those observed in murine models. Consequently, despite promising antitumor activity in some preclinical models, particularly colon cancer models in which PDE4 is also expressed by tumor cells, its clinical value as a neutrophil‐directed anticancer strategy remains unestablished.

Metabolic suppression of neutrophil‐associated immunosuppression has also been explored. Arginase‐1 (ARG1) reduces l‐arginine availability and thereby suppresses lymphocyte proliferation. The ARG1 inhibitor INCB001158 was evaluated as monotherapy and in combination with pembrolizumab in a Phase I study (NCT02903914), but the combination failed to demonstrate clear efficacy [175]. In contrast, a separate Phase I/II study combining INCB001158 with cytotoxic chemotherapy (NCT03314935) suggested that a subset of patients with advanced biliary tract cancer might benefit. These divergent findings suggest that metabolic targeting of immunosuppressive neutrophil‐associated pathways may depend on tumor type, treatment context, and the presence of complementary mechanisms capable of remodeling the suppressive TME.

NETs represent another potential therapeutic target. Recombinant human DNase I has been clinically used for cystic fibrosis and has been investigated in HNSCC (NCT00536952) to reduce the viscosity of tumor‐associated secretions. Although this study does not directly establish NET inhibition as an anticancer mechanism, it provides a translational framework for targeting extracellular DNA structures. PAD4 inhibition has similarly shown antitumor activity in preclinical models, but clinical validation remains unavailable. These approaches must balance suppression of tumor‐promoting NETs against preservation of their physiological antimicrobial functions.

#### 4.3.3 Phenotypic Reprogramming

Rather than eliminating TANs, phenotypic reprogramming aims to shift their function from tumor‐supportive toward tumor‐suppressive activity. N1‐like neutrophils can promote tumor cell killing and adaptive immune activation, whereas N2‐like populations are associated with ARG1 expression, immunosuppression, angiogenesis, and metastasis [176, 177]. This functional plasticity provides a potential advantage over indiscriminate neutrophil depletion because beneficial neutrophil functions may be retained.

TGF‐β represents a major regulator of TAN polarization. Inhibition of TGF‐β signaling has therefore been proposed to reduce immunosuppressive neutrophil programs and promote antitumor activity. However, because TGF‐β regulates multiple stromal and immune populations, the contribution of neutrophil reprogramming to the overall therapeutic response remains difficult to isolate clinically. Microbial and trained‐immunity‐based approaches provide alternative strategies for inducing cytotoxic neutrophil phenotypes. For example, bacterial bioparticles have been reported to induce IFN‐γ‐associated N1‐like neutrophil programs [178], whereas β‐glucan has been investigated as a means of inducing trained innate immune responses.

CD40 agonists and VISTA antagonists further illustrate the potential of functional reprogramming. CD40 agonists such as sotigalimab have shown immune activation and durable responses in selected cancer populations, including melanoma and pancreatic cancer, although clinical efficacy has been heterogeneous (NCT03214250 and NCT03123783). VISTA‐directed therapies are also being evaluated in combination with immune checkpoint inhibitors (NCT05082610). These observations suggest that neutrophil reprogramming may be particularly relevant when tumors retain sufficient immune competence to respond to functional conversion rather than cell depletion.

Collectively, current clinical and preclinical evidence indicates that no single neutrophil‐targeting strategy is universally applicable to cancer. Depletion may be effective when immunosuppressive neutrophils or PMN‐MDSCs dominate the TIME but risks eliminating beneficial populations. Recruitment blockade can limit tumor‐promoting infiltration but may be undermined by compensatory chemotactic pathways or insufficient target prevalence, as illustrated by the clinical experience with reparixin. Metabolic or effector inhibition may preserve neutrophil abundance but appears highly dependent on tumor biology and combination treatment. Phenotypic reprogramming offers greater potential to preserve beneficial functions but remains less clinically validated. These differences underscore the need for biomarker‐guided patient selection and disease‐specific therapeutic design rather than universal neutrophil suppression.

### 4.4 Limitations of Conventional Neutrophil Modulation Strategies

Although broad neutrophil‐targeting strategies have generated clinically meaningful advances, their therapeutic limitations become increasingly apparent when considered across infectious, inflammatory, and malignant diseases. The clinical experience summarized above indicates that the major challenge is not simply the lack of effective targets, but the difficulty of selectively modifying pathogenic neutrophil functions while preserving their physiological roles. First, limited cellular specificity remains a fundamental limitation. Many clinically used agents target signaling pathways or inflammatory mediators that are shared by neutrophils and other immune or tissue cells. Cytokine inhibitors, JAK inhibitors, PDE inhibitors, complement inhibitors, and CXCR4 antagonists therefore exert effects beyond neutrophils [74]. Although this broader activity can be therapeutically beneficial in systemic inflammation, it makes it difficult to distinguish neutrophil‐dependent efficacy from off‐target or parallel immunomodulatory effects. Second, the therapeutic window of neutrophil inhibition is often narrow. Neutrophils are essential for antimicrobial defense, tissue repair, and vascular homeostasis. Clinical experience with CXCR1/2 inhibition illustrates how reducing pathological recruitment can be accompanied by reductions in circulating neutrophils, potentially compromising host defense [142]. Similarly, complete inhibition of NET formation, protease activity, or oxidative responses may interfere with physiological antimicrobial functions [179]. Thus, the optimal intervention is unlikely to be maximal suppression but rather context‐dependent attenuation of pathological activity. Third, disease and patient heterogeneity strongly influence therapeutic response. The pathogenic contribution of neutrophils differs substantially between diseases and even between patients with the same disease. Increasing neutrophil abundance is appropriate for quantitative neutropenia but may be detrimental in neutrophil‐driven inflammatory disorders. In cancer, depletion may be beneficial when immunosuppressive PMN‐MDSCs dominate, whereas functional reprogramming may be preferable when antitumor neutrophil activity can be preserved. Clinical experiences with reparixin and INCB001158 further indicate that therapeutic efficacy may depend on the prevalence of the targeted population and the choice of combination therapy. Fourth, target‐pathology mismatch and compensatory pathways remain major causes of translational failure. Blocking a molecular pathway does not necessarily produce clinical benefit if that pathway is not a dominant driver of disease or if parallel pathways compensate for its inhibition. This problem is particularly relevant to chemokine‐mediated neutrophil recruitment, where multiple chemotactic signals can converge on the same cellular response. Similarly, differences between murine and human neutrophil biology may limit the predictive value of preclinical studies, as illustrated by the divergent effects of PDE4 inhibition [180].

These limitations have prompted increasing interest in disease‐associated neutrophil subsets as potential targets for more selective intervention. LDGs, enriched in systemic lupus erythematosus and other autoimmune diseases, exhibit enhanced spontaneous NET formation, cytokine production, and endothelial toxicity and are associated with disease activity. However, current approaches remain largely indirect, targeting LDG generation, recruitment, or effector functions through pathways such as GM‐CSF, CXCR1/2, JAK signaling, or NET formation, rather than selectively eliminating LDGs themselves. CD177 provides a more direct approach to neutrophil‐selective targeting. CD177 is expressed on a subset of circulating neutrophils and can serve as a surface handle for targeted drug delivery [181]. CD177‐binding peptides have been incorporated into NPs to selectively deliver siRNAs, antisense oligonucleotides, or neutrophil‐modulating agents to human and murine neutrophils, enabling transient regulation of inflammatory pathways such as C5a receptor signaling. More recently, CD177 silencing was reported to reduce neutrophil infiltration and tissue inflammation in preclinical models of NLRP3‐associated autoinflammatory disease. Nevertheless, CD177‐directed therapeutics have not yet entered established clinical development, highlighting the translational gap between neutrophil‐selective targeting and clinical application [182, 183].

In cancer, targeting the pathways that sustain pathological neutrophil states represents an alternative to direct subset depletion. TGF‐β is a key regulator of TAN polarization toward an immunosuppressive N2‐like phenotype and also influences macrophages, fibroblasts, epithelial–mesenchymal transition, and T‐cell function [184, 185]. Accordingly, TGF‐β blockade may indirectly suppress pathogenic TAN programs while remodeling the broader tumor microenvironment. Several TGF‐β‐directed agents, including fresolimumab, NIS793, SAR439459, and small‐molecule receptor inhibitors, have entered clinical evaluation. However, their clinical efficacy has generally been modest, and these agents are not selective for TANs. For example, the Phase III NIS793 study in metastatic pancreatic ductal adenocarcinoma (NCT04935359) has been completed, whereas the Phase II NIS793 study in metastatic colorectal cancer (NCT04952753) was terminated early because of an unfavorable benefit–risk profile following a halt in NIS793 treatment. These findings illustrate the translational challenge of targeting shared regulatory pathways rather than disease‐specific neutrophil subsets.

An emerging strategy is to selectively control the accumulation of senescent neutrophils by restoring their physiological clearance. CXCR4^+^CD62L^−^ neutrophils represent a senescent‐like population characterized by enhanced stress responses, NETosis, and prolonged survival, and their accumulation has been associated with tissue injury and disease progression. Although CXCR4 antagonists such as plerixafor and mavorixafor have reached clinical use for CXCR4‐related disorders, they broadly alter leukocyte trafficking and do not selectively target senescent neutrophils. More recently, pharmacological inhibition of the PGE2 receptor EP2 with PF‐04418948 was reported to restore tissue‐resident macrophage‐mediated clearance of senescent neutrophils and reduce CXCR4^+^ senescent neutrophil accumulation in aged mice. This approach highlights a distinct precision strategy that targets the pathological persistence of neutrophil subsets by restoring physiological clearance rather than broadly suppressing neutrophil function [186].

Collectively, these approaches illustrate a transition from broad neutrophil modulation toward subset‐informed precision intervention, encompassing indirect suppression of pathological programs, direct neutrophil‐selective targeting, pathway reprogramming, and restoration of physiological clearance. However, most remain at the preclinical or early clinical stage, and disease‐specific markers with sufficient selectivity and translational robustness are still lacking. Thus, future strategies should integrate cellular specificity, disease‐specific molecular endotyping, spatial control, and functional selectivity. Rather than globally inhibiting neutrophil abundance or activity, emerging approaches increasingly seek to selectively target disease‐associated neutrophil populations or exploit their intrinsic trafficking properties to deliver therapeutic cargos to pathological sites. This transition from broad neutrophil modulation to spatially and functionally controlled intervention provides the conceptual basis for neutrophil‐based precision delivery platforms discussed in the following section.

## 5 Neutrophil‐Based Precision Delivery Platforms: Overcoming the Limitations of Broad Modulation

Neutrophil‐based drug delivery systems represent a promising strategy for precision therapeutics. The central concept is to harness the intrinsic immune functions of neutrophils to overcome key limitations of conventional nanocarriers, including rapid clearance in vivo, insufficient targeting capability, and poor penetration across biological barriers. Importantly, these systems may also help address the major limitations of current neutrophil‐targeted therapies, which are largely constrained by broad‐spectrum and nonspecific interventions. By exploiting the natural trafficking behavior, inflammatory tropism, and functional heterogeneity of neutrophils, engineered delivery platforms offer the potential to selectively target disease‐associated neutrophil subsets or microenvironments, thereby reducing off‐target effects and enabling more precise immunomodulation. Over the past decade of research progress in NDDS, as illustrated in Figure 4A–C, researchers have developed a variety of technical strategies. These primarily encompass three core categories: living cell delivery systems, cell membrane‐coated NPs, and cell‐derived vesicles and exosomes. Table 5 and Figure 4D–G summarize current research on neutrophil‐based delivery systems.

**FIGURE 4:**
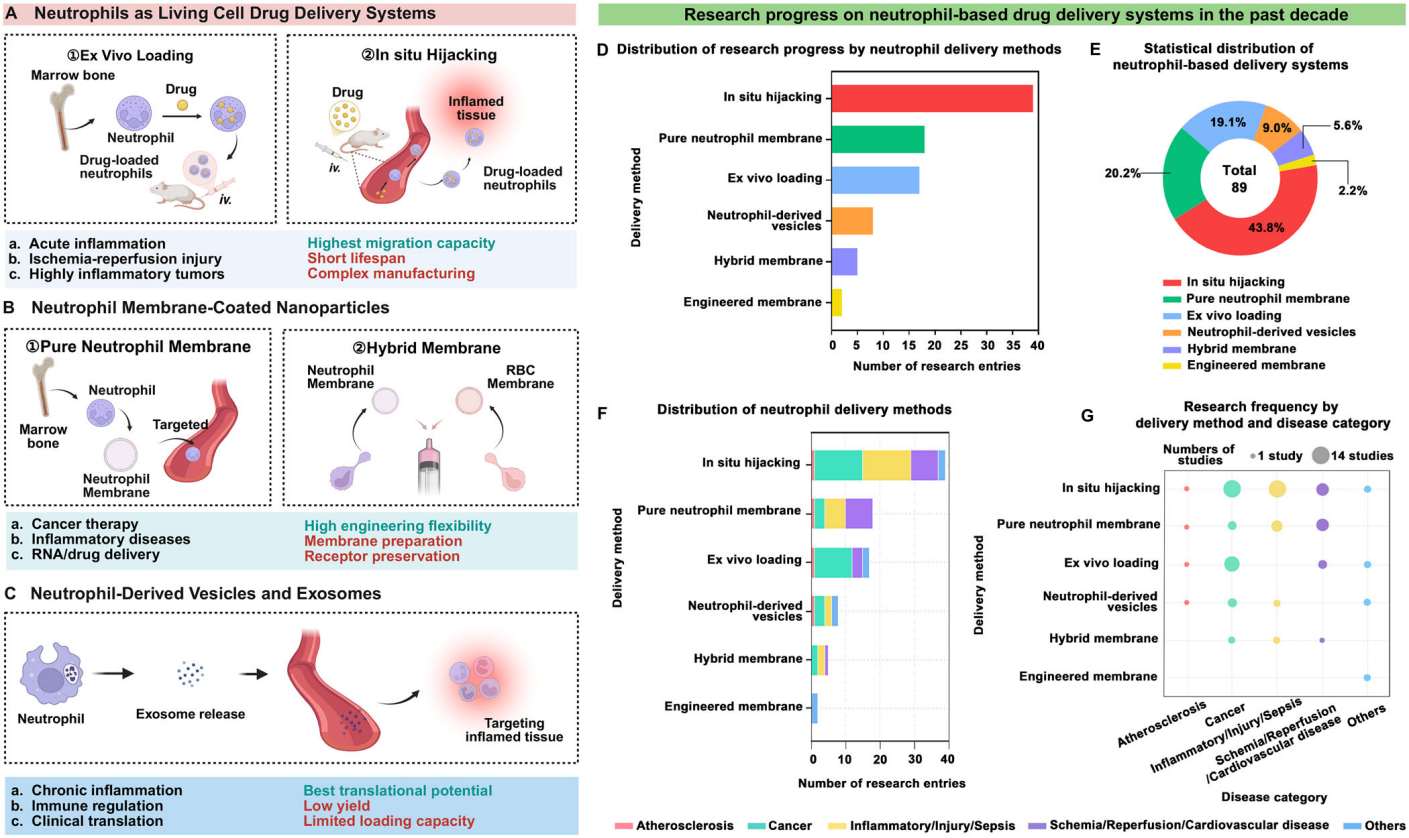
Neutrophil‐based drug delivery systems and their research landscape. (A) Neutrophils as living cell drug delivery systems, including ex vivo loading and in situ hijacking. (B) Neutrophil membrane‐coated nanoparticles, including pure neutrophil and hybrid membranes. (C) Neutrophil‐derived vesicles and exosomes as cell‐free delivery platforms. (D–G) Literature‐based analysis of research progress in neutrophil‐based drug delivery systems. PubMed was searched from January 1, 2020 to July 10, 2026, using the following strategy: (neutrophil [Title/Abstract]) AND (“drug delivery” [Title/Abstract] OR “neutrophil‐mediated” [Title/Abstract] OR “cell‐mediated delivery” [Title/Abstract] OR “neutrophil membrane” [Title/Abstract] OR “neutrophil‐derived vesicle” [Title/Abstract] OR “neutrophil‐derived exosome” [Title/Abstract] OR “neutrophil‐derived extracellular vesicle” [Title/Abstract] OR “membrane‐coated nanoparticle” [Title/Abstract] OR “in situ hijacking” [Title/Abstract]). After screening, a total of 89 articles were included in the analysis. Studies were assigned to a single primary delivery strategy and disease category; when multiple strategies or disease categories were reported, the primary strategy or disease context was used for classification to avoid double counting. (D) Distribution and (E) proportion of studies by delivery strategy. (F and G) Distribution of delivery strategies across disease categories.

**TABLE 5:** Research progress on neutrophil‐based drug delivery systems in the past decade.

Delivery method	NPs name	Disease models	Drug	Key mechanisms	References
Ex vivo loading and adoptive transfer	NPs@NEs	Osteoporosis indicators	CTX, PTH	Target bone marrow	[187]
	CAR–neutrophils@R‐SiO_2_	Glioblastoma	TPZ	Stem‐cell biotechnology	[188]
	PTX–CL/NE	Malignant glioma recurrence	PTX	Release NETs	[189]
	ND–MMSN	Glioma	Doxorubicin	Release NETs	[190]
	Abraxane/NEs	Gastric cancer	Abraxane	Release NETs	[191]
	AuNRBR/N	Lung cancer	AuNRBR	PTT	[192]
	SPIO–antiCD34/NEs	Myocardial ischemic injury	AntiCD34	Targeted EPCs	[193]
	MTX–liposome/NEs	IRI, tissue injury	MTX	Macrophage reuptake	[194]
	Neu‐Balloons	Atherosclerotic plaques	MiR‐126a‐5p	Bionic sonotargeting	[195]
	ZGO@TiO_2_@ALP‐NEs	Glioblastoma	PTX	Chemo/immuno	[196]
	DOX@BSA‐NEs	Breast cancer	Doxorubicin	Release NETs	[197]
	L‐P‐NPs@NEs	Myocardial ischemia–reperfusion injury	Luteolin	NET‐mediated ternary targeted delivery	[198]
	NEs@STING–Mal‐NP	TNBC	STING agonist	Activate the STING pathway	[199]
	NE+CAMP	Subcutaneous tumor	—	Systemic immunity	[200]
	CLTX‐T‐CAR hPSC‐NEs	Glioblastoma	Cas13d‐mediated gene knockdown	Modifying human pluripotent stem cells	[201]
	VNP–PD1 nb	Melanoma lung metastasis	VNP	Release NETs	[202]
	EM@MSNs‐NEs	—	DOX	Bacterial membranes mimic neutrophil micromotors	[203]
In situ hijacking of circulating neutrophils	PGP–PEG–DGL–PDP	MCAO	Catalase	Targeting CXCR2	[204]
	BSA‐NPs	Endometriosis	Glucose oxidase	BSA binds to neutrophils	[205]
	NPNs	EMT6 tumor	Cisplatin	Bacterial OMVs NPs bind to neutrophils, PTT	[206]
	CD11b–ANP	4T1 tumor	Decitabine	CD11b binds to neutrophils	[207]
	T‐TMP	Cerebral ischemia–reperfusion injury	Ligustrazine	CFLFLF bind to the FPR receptor of neutrophils	[208]
	OMV@PGZ	Ischemic stroke	Pioglitazone	Bacterial OMVs NPs bind to neutrophils	[209]
	OMVs/DOX	Glioma	DOX	Bacterial OMVs NPs bind to neutrophils	[210]
	cRGD‐NPs	66cl4 tumor	—	cRGD bind to α_v_β_3_ of neutrophils	[211]
	cRGD–PEG–DSPE	Cerebral ischemia	ER	cRGD targeting integrin αvβ1 of neutrophils	[212]
	T‐GSK	TBI	GSK484	CFLFLF bind to the FPR receptor of neutrophils	[213]
	Ac‐PGP–tFNA	Sepsis	Baicalin	Targeting CXCR2 of neutrophils	[214]
	aLy6G–IFNβ@TLP	Autoimmune encephalomyelitis	Tannic acid	Ly6G bind to neutrophils, NET formation	[215]
	APTS	MCAO	A151	TP targeted peptide bind to TGase	[216]
	n‐DOCPs	Acute lung injury	Dexamethasone	Hijacks neutrophils via CR3 phagocytosis.	[217]
	PMB@LNV–SyBV	Bacterial infections	Polymyxin B	Inflammation‐targeted fusion vesicles	[218]
	Ab/PTX NC	Lung cancer	PTX	Anti‐CD11b binds to neutrophils	[219]
	N‐in‐M	Intractable lung inflammation	Nexinhib20	Inhibits neutrophil recruitment and degranulation	[220]
	OMVs–DSPE–PEOz	B16‐F10 tumor	si‐CCDC25	OS‐D selectively binds neutrophils via PAMPs	[221]
	Dex@GNPs	Acute inflammation	Dexamethasone	Induce neutrophil apoptosis	[222]
	DNase‐I@PDA NPs, Siv@PLGA NPs	Pulmonary fibrosis	DNase‐I, Sivelestat	Targeting NETs and neutrophil elastase (NE)	[223]
	5HT‐NP@D, RLA	Tumor metastasis	DNase I, CPT	Degrades NETs, Blocks mitochondrial NETosis	[224]
	cLipo‐DC	Endometriosis	2‐DG, CQ	cFLFLF binds neutrophils via FPRs	[225]
	SA‐2@NCs	Breast cancer	Ibrutinib	PDT/PTT enhances neutrophil tumor infiltration	[226]
	DSPG/sLip	Acute otitis media	Ciprofloxacin	Applications of partially hydroxylated liposomes	[227]
	RPPT@PD/sV NCs	Myocardial ischemia–reperfusion injury	si‐VCAM‐1, dexamethasone	cRGD bind to α_v_β_3_ of neutrophils	[228]
	OMV@PCB NPs	4T1 tumor	Ce6, SB525334	PDT, Inducing the production of ROS to kill tumor cells	[229]
	cRGD–SVT–Lipo	Atherosclerosis	SVT	Inhibit NET formation	[230]
	PSN@T, FK@S	Sepsis	TGL, SVT	Platelet–neutrophil crosstalk	[231]
	SPPS	4T1 tumor	VNP20009	Inhibit NET formation	[232]
	APTs	Sepsis	tFNAs	Targeting CXCR2	[233]
	NT‐NPs	Thrombus	Hydroxychloroquine	NEBP binds to NE of neutrophil	[234]
	DOX–SAL	Rheumatoid arthritis	DOX	SA binds to l‐selectin of neutrophil	[235]
	DSPE–PEG‐c (RGDfk)	Multiple myeloma	BMS‐202	Interrupted the PD‐1/PD‐L1 axis	[236]
	NLP‐Vi/Vo	Acute myeloid leukemia	PLK1 inhibitor, vincristine	Neutrophil hitchhiking	[237]
	OMV@PDA	Breast cancer	—	EPR effect, photothermal therapy	[238]
	BTST NPs	Rheumatoid arthritis	Sivelestat	Block the release of NETs	[239]
	Fe/Al LDH–anti‐EphA2/Ly6G	Pancreatic cancer	Fe/Al LDH	Ly6G binds to neutrophil, tumor‐derived exosomes	[240]
	PC@PLGA	Acute lung injury	Doxorubicin	Programmed cell removal	[241]
	NA1‐loaded NPs	Stroke	Tat‐NR2B9c/NA1	Intracranial injection	[242]
Pure neutrophil membrane‐coated nanoparticles	DHA@ANeu–DDAB	Acute respiratory distress syndrome	Docosahexaenoic acid	First tissue, then cell	[243]
	NM‐NP–CFZ	Cancer metastasis	Carfilzomib	Continuously target CTCs in circulation	[244]
	GA@NMHOP	Acute myocardial infarction	18β‐Glycyrrhetinic acid	ROS‐responsive, neutrophil‐derived membrane vesicles	[245]
	MNM/siRNA NPs	Myocardial ischemia–reperfusion injury	siRNA	Neutrophil membranes modified by hemagglutinin (HA) and integrins	[246]
	Nm@MSNs	Non‐Hodgkin's lymphoma	Doxorubicin, Shanzhiside methylester	Induce apoptosis in tumor cells	[247]
	GCZM	4T1 tumor	Gox, CPO	GOx/CPO@ZIF‐8 generates HCl, NM‐coated for inflammation targeting.	[248]
	CEF–RvD1–NMVs	Bacterial infection	Ceftazidime	Targeting inflamed tissue	[249]
	As@ZM	Sepsis	Astragaloside IV	Reduce the release of NETs	[250]
	Neu‐LNPs	Myocardial infarction	TAPI‐1	Enhanced targeting of inflammatory tissue	[251]
	HG106‐ANM@Lip	Diabetic wounds	HG106	Apoptotic neutrophil membrane nanoparticles enhance efferocytosis and cellular uptake.	[252]
	N‐MVs	Ischemia–reperfusion injury	IL‐37	Reduce endothelial apoptosis and inflammation	[253]
	Neu MCs	Aortic dissection/aneurysm	tRF‐Gly‐CCC	Reduce the risk of blood vessel rupture	[254]
	AM@ZIF@NM	Atherosclerosis	miRNA‐155	NM targets plaque endothelium via CD18–ICAM‐1.	[255]
	POM@Mem	Intracerebral hemorrhage	POM nanozymes	Excellent ROS scavenging capacity	[256]
	Neu‐LP	Acute myocardial infarction	—	The homing ability of neutrophils in infarct tissue	[257]
	R4F‐NM@F127	Rheumatoid arthritis	Celastrol	R4F‐NM@F127 reprograms macrophages, inhibiting RA.	[258]
	RvD2‐HVs	Ischemic stroke	Resolvin D2	Specifically target inflamed brain endothelium during I/R	[259]
	KGF‐Neus	Inflammatory bowel diseases	Keratinocyte growth factor	Anti‐inflammatory properties	[260]
Hybrid membrane systems	PNMAuDIs	Metastatic breast cancer	DOX, indocyanine green	Platelet–neutrophil hybrid membrane	[261]
	RNP@PNM	4T1 tumor	R848, CD47	Platelet–neutrophil hybrid membrane	[262]
	Res‐PD‐L1@nmEVs	Lung ischemia–reperfusion injury	Resveratrol	Neutrophil membrane coated PD‐L1 hybrid vesicles	[263]
	TTob@NPM	Pseudomonas aeruginosa infection	Tobramycin	Anti‐inflammatory, toxin/bacteria‐eliminating, and immunomodulatory functions	[264]
	NM‐PB	Ulcerative colitis	Prussian blue	ROS removal/inhibition of cytokine–cytokine receptor interactions	[265]
Engineered membrane mimetics	NCCR	Corneal neovascularization	VEGF	Inhibits angiogenesis and inflammation	[266]
	GM@T‐NNPs	Intervertebral disc degeneration	Transforming growth factor‐beta 1	Inhibiting the PI3K–AKT signaling pathway	[267]
Neutrophil‐derived vesicles and exosomes	NEs‐Exos	Glioma	Doxorubicin	BBB penetration and tumor targetability	[268]
	VEGF–aPMNEM–ECM	Diabetic wound	VEGF	Antibacterial activity and wound‐healing effects	[269]
	piRNA‐17560	Breast cancer	Docetaxel	Exosomes secreted by ageing neutrophils	[270]
	LAND‐Vs	Pneumonic inflammation	CD55	CD55+ LAND‐Vs suppress inflammation via complement inhibition.	[271]
	NNVs	HGC27 tumor	DOX	Apoptosis induced via the caspase pathway	[272]
	NMVs	Atherosclerosis	miR‐155	Enhanced activation of NF‐κB	[273]
	NDEVs	Cytokine storm syndrome	miR‐27a‐3p	Suppressed the expression of Suclg1/regulation of macrophage activation	[79]
	BD PMN exosomes	Behçet's disease	miR‐122‐5p	Inhibit macrophage activation	[274]

Abbreviations: ANM, apoptotic neutrophil membrane; BBB, blood–brain barrier; BSA, bovine serum albumin; CAMP, cationic antimicrobial peptide; CAR, chimeric antigen receptor; Ce6, Chlorin e6; CPT, camptothecin; CTX, cyclophosphamide; DOX, doxorubicin; EPR, enhanced permeability and retention effect; FPR, formyl peptide receptor; GNPs, gold nanoparticles; Gox, glucose oxidase; IRI, ischemia–reperfusion injury; LDH, layered double hydroxide; MCAO, middle cerebral artery occlusion; MMP‐9, matrix metalloproteinase‐9; MSNs, mesoporous silica nanoparticles; Nes, neutrophil exosomes/neutrophil‐derived vesicles; NETs, neutrophil extracellular traps; Neu, neutrophil; NM, neutrophil membrane; NMs/NMVs, neutrophil‐derived membrane vesicles; OMVs, outer membrane vesicles (bacterial); PAMPs, pathogen‐associated molecular patterns; PDA, polydopamine; PDT, photodynamic therapy; PLGA, poly(lactic‐co‐glycolic acid); PTH, parathyroid hormone; PTT, photothermal therapy; PTX, paclitaxel; RGD/cRGD, arginylglycylaspartic acid (cyclic) peptide; SA, sialic acid; SPIO: superparamagnetic iron oxide; TBI, traumatic brain injury; TNBC, triple‐negative breast cancer; TPZ, tirapazamine; VEGF, vascular endothelial growth factor; ZIF‐8, zeolitic imidazolate framework‐8.

### 5.1 Neutrophils as Living Cell Drug Delivery Systems

Directly employing intact and functionally active neutrophils as drug carriers represents one of the most cutting‐edge “living therapeutics” in the biomedical field. Compared with other cellular carriers, such as erythrocytes and macrophages, neutrophils exhibit remarkable motility within the circulation and can dynamically migrate along inflammatory gradients. This strategy not only preserves the full repertoire of native receptors on the cell membrane but also exploits the intrinsic ability of neutrophils to actively traverse biological barriers, including the BBB, and penetrate dense tissues, thereby enabling a “Trojan horse” like precision drug delivery.

#### 5.1.1 Biological Advantages of Neutrophils as Living Cell Drug Delivery Systems

In contrast to conventional nanocarriers that rely primarily on passive targeting mechanisms, neutrophils actively participate in pathological processes, enabling precise and efficient delivery of therapeutic payloads. A defining advantage of neutrophils lies in their intrinsic inflammatory tropism and active homing capability. In response to chemotactic gradients generated at sites of infection, tumor growth, or tissue injury, neutrophils are rapidly recruited from the bloodstream into affected tissues [275]. This endogenous targeting mechanism allows drug‐loaded neutrophils to navigate toward inflammatory microenvironments or deep tumor regions, overcoming the limitations associated with passive accumulation strategies such as the enhanced permeability and retention effect. Neutrophils are also uniquely capable of traversing biological barriers. During inflammatory activation, they undergo transendothelial migration and infiltrate tissues, and under certain pathological conditions, they can penetrate the BBB [276]. This property provides a viable route for delivering therapeutics to otherwise inaccessible sites, including the CNS, which remains a major challenge for traditional delivery platforms. As endogenous cells, neutrophils exhibit favorable biocompatibility and circulation behavior. Their intrinsic compatibility with the host immune system reduces the risk of rapid clearance and adverse immunogenic responses commonly observed with synthetic carriers. The use of autologous neutrophils further enhances safety and supports translational feasibility. Another key feature is their capacity for stimulus‐responsive cargo release. Upon reaching target tissues, neutrophils can release therapeutic payloads through multiple physiological processes, including degranulation, apoptosis, and NET formation [277]. These mechanisms enable localized and controlled drug release, thereby improving therapeutic efficacy while minimizing systemic exposure. In addition, direct interactions between neutrophils and surrounding cells may facilitate efficient transfer of therapeutic agents to target cell populations. Beyond their role as carriers, neutrophils contribute to immune modulation [74]. Through interactions with macrophages, DCs, and T cells, they can influence the local immune microenvironment, potentially amplifying therapeutic outcomes. In the context of cancer, such interactions may enhance antitumor immunity by reshaping the tumor immune landscape. Finally, neutrophil‐based delivery systems offer considerable engineering flexibility. Strategies such as ex vivo loading, surface functionalization, and in situ hijacking enable precise modulation of neutrophil behavior and payload delivery [140], allowing the system to be tailored for specific therapeutic applications.

Collectively, neutrophils integrate active targeting, barrier penetration, biocompatibility, and immunomodulatory capacity into a single platform, positioning them as a promising modality for drug delivery in cancer, infectious diseases, and CNS disorders. Nonetheless, challenges including their short lifespan, phenotypic plasticity, and scalability of clinical implementation remain to be addressed.

#### 5.1.2 Ex Vivo Loading and Adoptive Transfer

Ex vivo loading of neutrophils (NEs) represents a direct and extensively investigated strategy within NDDSs, where freshly isolated neutrophils serve as living carriers for therapeutic cargoes. This approach exploits the innate migratory and inflammatory homing capabilities of neutrophils while allowing for precise control over drug loading prior to systemic reinfusion. Typically, neutrophils are isolated from peripheral blood via density gradient centrifugation or immunomagnetic separation to ensure high purity and viability. Given their transient lifespan and extreme sensitivity to external stimuli, meticulous handling is imperative to minimize unintended activation during isolation, as premature activation can compromise their chemotactic efficiency and in vivo trafficking behavior. Drug loading is achieved through several mechanisms, among which phagocytosis is the most prevalent for NP‐based formulations due to the natural internalizing capacity of neutrophils [278]. Alternatively, small‐molecule drugs can be introduced through passive diffusion or membrane permeabilization, while surface conjugation allows for the attachment of therapeutic agents without disrupting intracellular homeostasis. The selection of a loading methodology depends on the physicochemical properties of the cargo and the intended release profile.

In representative studies, Xue et al. [189] developed paclitaxel cationic liposomes (PTX–CL)/NEs by incubating neutrophils isolated from mouse bone marrow with PTX–CL in vitro. These cells were subsequently reinfused into mice for the treatment of postoperative glioma. In vitro simulations indicated no significant drug leakage from PTX–CL/NEs under physiological conditions within 8 h. Following reinfusion, in vivo imaging demonstrated that the fluorescence intensity in the cranial region of the PTX–CL/NE group was significantly higher than that of other groups, confirming that PTX–CL/NEs maintain functional integrity and can cross the BBB into tumor tissues under cytokine induction. Furthermore, Wang et al. developed a biomimetic nanopathogen (NPN) system for neutrophil‐targeted NP delivery, leveraging the natural propensity of neutrophils to recognize and engulf pathogens such as bacteria. Specifically, NPs were coated with bacterial outer membrane vesicles (OMVs) to prepare NPNs. The diverse PAMPs present on OMVs are sensed by neutrophils, mediating efficient phagocytosis. Neutrophils carrying NPNs actively migrate via chemotaxis toward inflammatory tumor sites following photothermal therapy (PTT). Upon stimulation by inflammatory molecules at the site, the neutrophil membranes rupture, releasing the NPNs. The released NPNs, loaded with cisplatin, effectively eliminate residual tumor cells, working synergistically with PTT to achieve complete tumor eradication. This strategy is broadly applicable for the neutrophil‐specific targeting of various nano‐ or microparticles and can be extended to the treatment of multiple diseases involving neutrophil infiltration, including tumors and inflammatory disorders [206]. To advance the clinical translation of this strategy, Ju et al. utilized density gradient centrifugation to isolate human peripheral blood neutrophils and loaded them with paclitaxel albumin NPs (abraxane) to construct abraxane/NEs. When reinfused into SNU‐719 nude mouse models, abraxane/NEs combined with local radiotherapy significantly improved therapeutic outcomes and intratumoral drug distribution compared with the abraxane group, without inducing adverse reactions in the mice [191].

A critical consideration in this strategy is the preservation of neutrophil functionality postloading. Excessive cargo burden or harsh loading conditions may impair cell viability, reduce migratory capacity, or trigger premature degranulation. Consequently, optimizing the balance between loading efficiency and cellular integrity is essential. Engineered NPs can also be designed to remain inert during circulation, releasing their payload only upon exposure to inflammatory stimuli such as ROS, acidic pH, or specific enzymes at the disease site. Upon reinfusion, drug‐loaded neutrophils retain their ability to navigate chemotactic gradients toward inflamed or diseased tissues. At the target site, the therapeutic cargo is released through mechanisms such as cell activation, apoptosis, or the formation of NETs, enabling localized delivery with reduced systemic toxicity.

The ex vivo loading and reinfusion strategy for NEs shares conceptual similarities with CAR‐T therapy, offering a novel paradigm for oncological treatment. However, its clinical feasibility and safety require comprehensive evaluation. Neutrophils are characterized by a relatively short lifespan, although estimates of their circulatory kinetics vary depending on the experimental approach. An in vivo labeling study reported an average circulatory lifespan of approximately 5.4 days under homeostatic conditions, whereas subsequent kinetic analyses estimated a blood half‐life of less than 1 day. Ex vivo, isolated neutrophils generally survive for only a few hours [279]. This necessitates that isolation, loading, and reinfusion be completed within a highly constrained timeframe [19]. Moreover, maintaining NE viability during isolation is paramount; damaged NEs may express “find‐me” and “eat‐me” signals, triggering clearance by phagocytes and leading to significant off‐target effects. Achieving efficacy thus requires refined isolation and loading techniques, alongside robust viability assays for prereinfusion quality control. Finally, the challenge of large‐scale neutrophil sourcing must be addressed. If autologous NEs are utilized, potential heterogeneity between patient‐derived and healthy donor cells must be considered, warranting the establishment of NE subtyping for individualized therapy. Conversely, the use of allogeneic NEs necessitates a thorough assessment of risks associated with immune rejection.

#### 5.1.3 In Situ Hijacking of Circulating Neutrophils

The in situ hijacking strategy, frequently termed in vivo hitchhiking, involves the systemic administration of nanotherapeutics designed to spontaneously associate with endogenous neutrophils within the bloodstream [276]. By bypassing ex vivo manipulations, this approach overcomes critical translational hurdles, including the limited 7‐h circulatory half‐life and premature cellular activation typically associated with adoptive cell transfer. This noninvasive paradigm leverages the intrinsic inflammatory tropism and the innate ability of neutrophils to actively traverse biological barriers, such as the BBB, to achieve the precise delivery of therapeutic payloads into deep‐seated inflammatory, tumoral, or damaged tissues.

Recent advances in molecular engineering have introduced new approaches to improve hijacking efficiency. One such advancement is “voluntary opsonization,” which repurposes the protein corona into a targeting facilitator [217]. By utilizing liposomes composed of inverse phosphocholine lipids (DOCP), researchers triggered the rapid enrichment of the complement fragment iC3b on the NP surface. This iC3b layer serves as a high‐affinity natural ligand for complement receptor 3 (CR3), which is significantly upregulated on neutrophils during acute inflammation. In models of ALI, this corona‐driven hijacking enabled drug‐laden neutrophils to penetrate the alveolar‐capillary barrier within hours, effectively eliminating intraparenchymal bacteria [206].

To further address kinetic constraints imposed by high blood flow velocity, an “endothelial deceleration” strategy was developed using the APTS nanoplatform [216]. This system employs a targeted peptide (TP) that anchors to transglutaminase (TGase) expressed on inflamed vascular walls, thereby creating a docking interface that enhances NP capture during neutrophil rolling and adhesion. In combination with sialic acid (SA) modification targeting l‐selectin (CD62L), this approach achieved a 1.7‐fold increase in hijacking efficiency. In IS models, this strategy resulted in a fourfold enhancement in brain drug delivery. Notably, these hijacked NPs also scavenged ROS, reprogramming neutrophil fate from proinflammatory NETosis toward apoptosis, and subsequently facilitating cargo transfer to microglia, leading to a 5.1‐fold reduction in infarct volume.

Importantly, beyond circulating neutrophils, recent studies have expanded the concept of in situ hijacking to brain‐adjacent immune niches. For example, Gao et al. [242] demonstrated that drug‐loaded NPs can hijack calvarial immune cells via intracalvariosseous administration, enabling their migration through skull‐meninges microchannels directly into the CNS, thereby bypassing the BBB entirely. These NP‐loaded immune cells exhibited rapid, lesion‐directed trafficking and achieved highly efficient delivery to ischemic brain regions, significantly improving both acute and long‐term outcomes in stroke models. To evaluate the clinical translatability of this strategy, researchers conducted the SOLUTION trial (NCT05849805), a prospective, randomized, open‐label clinical trial involving patients with malignant middle cerebral artery infarction. Real‐world evidence from this study confirms the feasibility and safety of immune cell‐mediated delivery, providing a robust foundation for the translational application of these vectors in treating neurovascular diseases. This work highlights an alternative “local in situ hijacking” paradigm, in which immune cells residing in brain‐bordering niches are directly exploited as delivery vectors, further expanding the versatility of immune cell‐mediated transport.

In summary, in situ hijacking of endogenous immune cells represents a transformative paradigm in drug delivery, effectively converting circulating or tissue‐resident immune cells into autonomous, programmable carriers within the body. Its success relies on three key engineering principles: molecular specificity, kinetic synchronization, and fate reprogramming. By integrating these elements, modern nanoplatforms not only enhance targeting precision but also actively modulate the biological behavior of carrier cells. While this strategy shows exceptional promise for accessing sanctuary sites such as the brain parenchyma and deep tumor cores, future studies must focus on maintaining payload stability without compromising cellular homing functions. Consequently, therapeutic strategies based on emerging drug delivery systems utilizing neutrophil‐mediated hitchhiking hold great promise for the treatment of inflammatory diseases and malignant tumors. Further advances in drug delivery technology may improve the targeting and delivery performance of neutrophil‐based hitchhiking systems. Although the clinical application of neutrophil‐hitchhiking strategies still faces numerous challenges, they represent a new therapeutic direction for the treatment of these diseases.

#### 5.1.4 Artificial Neutrophil‐Mimicking Systems

The development of artificial neutrophil (AN)‐mimicking systems represents a pivotal advancement in biomimetic engineering, aiming to circumvent the inherent limitations of natural neutrophils, such as their short physiological half‐life and the challenges associated with large‐scale in vitro expansion. The conceptual foundation for these systems rests on the principle that exosomes derived from immune cells possess biological functions similar to their source cells, primarily regulating systemic inflammation and immune responses while playing critical roles in immune activation, antigen presentation, and metabolic regulation. By integrating the targeting capabilities and functional proteins of neutrophils into synthetic scaffolds, researchers have created sophisticated platforms capable of navigating complex physiological environments and delivering therapeutic payloads with high precision. These artificial constructs often utilize cell membrane camouflage to inherit the homing behavior and “do not eat me” signaling of native leukocytes, while incorporating engineered cores to provide spatiotemporal control over drug release and catalytic activity.

Recent breakthroughs have demonstrated the versatility of these systems across various infectious and oncological contexts. Jiang et al. developed micrometer‐sized ANs designed specifically to address vascular graft infections [280]. Their design integrates a metal‐organic framework (ZIF‐8) encapsulating glucose oxidase and chloroperoxidase within a DNA‐based coacervate framework, which is then enclosed in a phospholipid bilayer carrying catalase. A primary highlight of this study is the attainment of active chemotaxis: the ANs sense H_2_O_2_ gradients at inflammation sites and migrate autonomously toward the infection, where they catalyze the production of HOCl to eradicate Staphylococcus aureus, demonstrating a functional lifespan and stability far exceeding those of natural granulocytes.

In the field of neuro‐oncology, Deng et al. designed X‐ray‐activated nanoneutrophils (SPNENE) for orthotopic glioma immunotherapy [281]. This system consists of semiconducting polymer NPs and elastase embedded in a singlet oxygen‐responsive liposome, subsequently camouflaged with a neutrophil cell membrane. The study highlights that the neutrophil membrane facilitates BBB crossing and deep tumor infiltration; upon localized X‐ray irradiation, the semiconducting polymer generates singlet oxygen to trigger the release of elastase, which proteolyzes the CD95 death domain of tumor cells and induces immunogenic cell death (ICD), thereby activating a robust systemic adaptive T‐cell response.

Further expanding the application to metastatic disease, Niu et al. developed a neutrophil‐mimicking nanomedicine (NM@PLGA–MTI–OXA) to target colorectal cancer and its liver metastases [282]. Their design utilizes PLGA NPs to coencapsulate metronidazole and oxaliplatin, which are then coated with murine bone marrow‐derived neutrophil membrane vesicles. The research highlights the nanomedicine’ s dual‐targeting capability, which enables the effective elimination of tumor‐resident Fusobacterium nucleatum a key driver of chemoresistance while simultaneously reversing the EMT of tumor cells and remodeling the immunosuppressive microenvironment without disrupting the host's intestinal microbiota. Collectively, these artificial systems demonstrate that the integration of source‐cell membrane proteins, such as integrins and chemokine receptors, allows for highly efficient disease homing and therapeutic efficacy that mirrors and enhances the natural defensive roles of the innate immune system.

#### 5.1.5 Challenges Associated With Living Cell Drug Delivery Systems

Despite their intrinsic targeting and trafficking capabilities, living neutrophils should not be considered straightforward drug carriers. Their short lifespan, dynamic activation state, and diverse cell‐death pathways introduce several challenges that can directly affect cargo delivery and therapeutic safety.

Cargo loading and preservation of cellular function remain major challenges. Therapeutic cargos can be loaded onto the cell surface or into the intracellular compartment. However, excessive loading or harsh loading procedures may impair membrane integrity, viability, chemotaxis, and other neutrophil functions. Conversely, insufficient loading may result in inadequate therapeutic efficacy. Therefore, an appropriate balance between cargo loading and preservation of neutrophil function is essential [283].

The short lifespan of neutrophils further limits their use as carriers. Neutrophils must retain sufficient viability and functional activity throughout isolation, cargo loading, circulation, and target accumulation. Short‐lived cells require rapid processing after isolation to minimize loss of activity, highlighting the need for optimized preparation and quality‐control procedures [284].

The fate of cargo following neutrophil apoptosis or NETosis is another critical concern. Although neutrophil death at the target site could facilitate cargo release, premature apoptosis or NETosis may cause uncontrolled release during circulation, reducing targeting efficiency and increasing off‐target exposure. Thus, controlled release mechanisms are needed to synchronize cargo release with neutrophil activation or death at the disease site [285].

Premature neutrophil activation may compromise the safety of neutrophil‐based drug delivery systems. Exposure to inflammatory signals or the carrier itself may activate neutrophils before they reach the target tissue, triggering degranulation, ROS production, NET formation, and premature cargo release. Importantly, excessive NET formation may further aggravate pathological inflammation, as PAD4 released by neutrophils can generate autoantigens, while NETs can present these autoantigens together with danger signals and promote severe autoimmune responses [286, 287]. In addition, NETs can serve as scaffolds for platelet‐mediated thrombosis and contribute to inflammation under pathological conditions. Thus, although the responsiveness of neutrophils to the surrounding microenvironment provides an inherent advantage for targeted delivery, premature or excessive activation may conversely lead to uncontrolled cargo release, off‐target inflammation, thrombosis, and tissue injury.

Finally, the in vivo fate and clinical reproducibility of neutrophil‐based delivery remain insufficiently characterized. It is necessary to determine whether loaded neutrophils reach the target tissue while retaining their cargo and when and where cargo release occurs. Improved tracking technologies and standardized quality‐control parameters, including viability, activation state, chemotactic function, and cargo retention, are therefore required for clinical translation [7].

Overall, future studies should focus on minimally disruptive cargo loading, preservation of neutrophil function, controlled release following target‐site activation or cell death, and comprehensive monitoring of cell and cargo fate. These advances are essential for transforming neutrophils from promising biological carriers into predictable and clinically translatable drug‐delivery systems. Recent work on cell–drug conjugates similarly emphasizes that the biological functions of living cells provide unique delivery advantages while simultaneously introducing additional translational complexity.

### 5.2 Neutrophil Membrane‐Coated NPs

Cell‐membrane‐coated NPs represent a sophisticated biomimetic strategy for targeted drug delivery, emerging in recent years as a versatile nanoplatform for multifaceted biomedical applications. By leveraging membranes derived from diverse cell lineages including cancer cells, platelets, erythrocytes (RBCs), stem cells, and immune cells these systems can sequester the essential structural motifs and functional proteins of their parent cells, thereby preserving specific biological traits. For instance, cancer cell membranes express tumor‐specific adhesion molecules that facilitate homologous targeting and homing to primary tumor sites. Conversely, erythrocyte‐ and platelet‐derived membranes are enriched with CD47, a “self‐recognition” signal that effectively subverts phagocytic clearance and prolongs systemic circulation [288]. The preparation of cell‐membrane‐coated NPs generally involves membrane isolation followed by membrane–NP fusion, which can be achieved using extrusion, sonication, electroporation, or microfluidic technologies. These approaches facilitate the formation of a continuous membrane coating around NP cores while preserving key membrane proteins and their associated biological functions [285, 289]. Building upon this biomimetic paradigm, neutrophil‐membrane‐camouflaged nanomaterials integrate the innate inflammatory tropism of neutrophils with the tunable characteristics of synthetic nanocarriers, significantly enhancing both the circulatory stability and the site‐specific accumulation of drug delivery systems at neoplastic tissues.

#### 5.2.1 Biological Advantages of Neutrophil Membrane Coating

Neutrophil membrane coating endows synthetic NPs with a unique set of biological functions that closely mimic the behavior of native neutrophils, thereby offering significant advantages for targeted drug delivery. By inheriting the complex membrane protein repertoire of neutrophils, these biomimetic nanocarriers can actively participate in physiological and pathological processes, rather than merely serving as passive delivery vehicles. One of the most prominent advantages is inflammatory tropism [290]. Neutrophil membranes retain adhesion molecules such as l‐selectin (CD62L), β2 integrins, and chemokine receptors, which enable coated NPs to recognize and respond to inflammatory signals. This allows the nanocarriers to follow chemotactic gradients and preferentially accumulate at sites of inflammation, including tumors, infections, and ischemic tissues. Such active targeting significantly enhances delivery efficiency compared with conventional NPs [291]. Another key feature is the ability to interact dynamically with the vascular endothelium [292]. Neutrophil membrane‐coated NPs preserve the multistep adhesion cascade characteristic of neutrophils, including rolling, adhesion, and transmigration. Through interactions with endothelial selectins and intercellular adhesion molecules, these nanocarriers can adhere to activated endothelium and potentially penetrate biological barriers, such as the BBB, under pathological conditions [293].

Collectively, neutrophil membrane coating integrates targeting, immune modulation, and biological responsiveness into a single platform. These advantages position it as a powerful strategy for precision nanomedicine, particularly in diseases where inflammation plays a central role, such as cancer, cardiovascular disorders, and CNS pathologies.

#### 5.2.2 Classification of Neutrophil Membrane‐based Systems

##### 5.2.2.1 Pure Neutrophil Membrane‐Coated NPs

Pure neutrophil membrane‐coated NPs (PNM‐NPs) represent a sophisticated paradigm in biomimetic drug delivery, eschewing traditional whole‐cell or hybrid membrane strategies in favor of high‐purity neutrophil plasma membranes. This approach ensures the maximal preservation of the native conformations and biological functionalities of critical surface receptors, such as CD11b/CD18, l‐selectin, and Fcγ receptors. These NPs possess a unique constellation of properties, including prolonged circulation, active inflammatory targeting, multivalent immunomodulation, and detoxification capabilities, which confer distinct therapeutic advantages in the treatment of inflammatory diseases, autoimmune disorders, malignancies, and infectious conditions. The successful fabrication of PNM‐NPs is predicated on the acquisition of high‐quality membrane fractions. Established methodologies include: (i) density gradient centrifugation for the isolation of primary neutrophils from peripheral blood or bone marrow, or the induced differentiation of myeloid cell lines such as HL‐60 [294]; (ii) hypotonic lysis coupled with mechanical disruption, employing gentle osmotic stress followed by nitrogen cavitation or ultrasonication to compromise membrane integrity; and (iii) differential centrifugation and density gradient purification typically using sucrose or Percoll to remove mitochondria, nuclei, and cytosolic components, thereby yielding high‐purity plasma membrane vesicles [295]. These purified membranes are subsequently assembled onto various synthetic templates such as polymeric cores, mesoporous silica, gold, or magnetic NPs via coextrusion or ultrasonic fusion to generate biomimetic core–shell architectures. Through the optimization of membrane‐to‐core mass ratios, extrusion cycles, and pore dimensions, researchers can precisely calibrate coating integrity, particle uniformity, and surface receptor density. In the context of engineering and clinical translation, the potency of PNM‐NPs is fundamentally tied to the high‐fidelity translocation of membrane proteins. A landmark study by Zhao et al. exemplified this potential by utilizing plasma membranes derived from differentiated HL‐60 cells to camouflage ROS‐responsive polyprodrug NPs for IS therapy [296]. The high‐fidelity preservation of essential membrane proteins, such as integrin β2, PECAM‐1, PSGL‐1, and VLA‐4, enabled these nanomedicines to navigate the systemic circulation and home toward ischemic lesions in MCAO mouse models. This targeted approach achieved a 15.2‐fold increase in therapeutic accumulation within the brain parenchyma compared with free drug administration, effectively reprogramming microglia toward anti‐inflammatory phenotypes while significantly mitigating systemic cardiovascular side effects. Furthermore, recent advancements have expanded the scope of PNM‐NPs through precision engineering of the donor membranes to achieve hierarchical or tissue‐specific targeting. For instance, Li et al. developed engineered nanovesicles (DHA@ANeu–DDAB) for ARDS by fusing activated neutrophil membranes enriched with high‐affinity β2 integrins via divalent cation induction with lung‐targeting lipids [243]. This “tissue first, cell second” strategy enabled the particles to first accumulate in lung tissue and then specifically bind to ICAM‐1/2 on inflamed vascular endothelium, blocking native neutrophil infiltration while delivering docosahexaenoic acid (DHA) to promote inflammation resolution. Similarly, Jiang et al. designed nanocomplexes (MNM/siRNA NPs) for MIRI therapy by coating siRNA‐loaded cationic polymer cores with neutrophil membranes engineered to overexpress hemagglutinin (HA) and integrin α9β1 [246]. The integrated integrins ensured precise homing to ischemic cardiac tissue, while the HA protein facilitated endosomal escape, allowing efficient silencing of integrin α9 in target neutrophils to reduce NET and microthrombus formation. The ability of these systems to leverage neutrophil‐specific effector mechanisms, such as burst release in pathological microenvironments, significantly enhances intratumoral drug distribution and the overall therapeutic index while mitigating the systemic toxicities associated with conventional delivery platforms. From an immunological and biosafety perspective, PNM‐NPs offer robust multivalent benefits. The presentation of the “marker‐of‐self” protein CD47 on the membrane surface allows the NPs to communicate with the SIRPα receptor on macrophages, transmitting inhibitory signals that attenuate sequestration by the reticuloendothelial system in the liver and spleen, thereby extending the circulatory half‐life. Beyond serving as specialized transport vehicles, PNM‐NPs act as active “cytokine sponges” and immunomodulators. For example, Ye et al. developed apoptotic neutrophil membrane (ANM)‐modified liposomes (HG106‐ANM@Lip) to promote diabetic wound healing [252]. By inheriting “eat me” signals like phosphatidylserine from apoptotic neutrophils, these NPs were preferentially recognized and internalized by DCs, synergistically activating the MERTK/AXL pathway to enhance the efferocytosis of apoptotic cells and remodel the inflammatory microenvironment. Furthermore, membrane receptors inherited on PNM‐NPs act as decoys to capture and neutralize excessive proinflammatory mediators such as TNF‐α and IL‐6 within the local milieu. This action effectively halts pathological inflammatory cascades and facilitates the restoration of physiological homeostasis. By integrating precision targeting, sustained bioavailability, and comprehensive immune modulation, this biomimetic platform establishes a transformative paradigm for precision therapy across diverse disease landscapes.

##### 5.2.2.2 Hybrid Membrane Systems

Biomimetic NDDSs compensate for the deficiencies of traditional platforms by endowing nanocarriers with properties such as prolonged circulation and immune evasion, thereby enhancing accumulation at pathological sites. However, biomimetic nanocarriers constructed from a single cell membrane type often possess restricted functionality that cannot fully satisfy the diverse requirements of modern biomedicine. Hybrid membrane systems resolve these limitations by integrating the distinct biological functions of various cell membranes into a single unified biomimetic platform. This strategy allows the resulting system to exhibit synergistic cross features derived from multiple parent cells, which significantly enhances the therapeutic potential of the carrier and expands the available repertoire of biomimetic tools. Such advancements offer greater flexibility and precision for the personalized treatment of malignancies and other complex disorders. For instance, platelet neutrophil hybrids combine the long circulatory profile and bacterial binding capacity of platelets with the robust inflammatory chemotaxis of neutrophils. Zhang et al. demonstrated this by coating antibiotic loaded framework nucleic acids with a neutrophil platelet fused membrane to treat Pseudomonas aeruginosa infections [264]. This platform utilized neutrophil like chemotaxis for site specific targeting and platelet like adhesion for docking with pathogens while simultaneously neutralizing toxins. Furthermore, hybridizing membranes allows for the orchestration of multivalent responses to penetrate complex biological barriers. For instance, leveraging the pathophysiological recruitment of platelets and neutrophils to postsurgical wound sites, Sheng et al. developed a twin‐drive precise delivery system [262] utilizing platelet–neutrophil hybrid membranes loaded with R848 and modified for CD47 blockade. This platform exploits the innate chemotactic tendencies of its parent cells to achieve targeted accumulation at tumor sites. Guided by fluorescence imaging, the system facilitates the localized reprogramming of M2 macrophages into the M1 phenotype and promotes DC maturation. By synergizing CD47‐mediated phagocytosis enhancement with T‐cell‐driven cellular immunity, this strategy establishes a cell‐specific response tailored to the inflammatory microenvironment, effectively inhibiting postsurgical recurrence and metastasis while significantly prolonging survival. Similarly, the integration of neutrophil and macrophage membranes has been applied to treat chronic conditions as shown by Hu et al. who engineered a hybrid membrane coated Prussian blue nanozyme for UC [265]. This system utilized the combined surface protein repertoire of both parent cells to enhance recruitment to inflamed mucosa and facilitate a reparative shift in the local immune microenvironment. These hybrid paradigms, including potential cancer neutrophil systems that stack homotypic targeting with inflammatory homing, establish a versatile framework for achieving multi target intervention in heterogeneous disease environments.

##### 5.2.2.3 Engineered Membrane Mimetics

The emergence of engineered neutrophil‐membrane‐based biomimetic systems represents a critical shift from basic membrane coating toward precision therapeutic interventions. These platforms utilize covalent or noncovalent modifications to integrate specific ligands onto the membrane surface, or are engineered as “cytokine sponges” to actively modulate complex inflammatory microenvironments.

Ligand‐modified membranes enhance the innate targeting capabilities of neutrophils by incorporating functional molecules, such as antibodies or therapeutic proteins, onto the membrane surface. For instance, Liu et al. developed a neutrophil–nanovesicle‐based eye drop (NCCR) designed for the treatment of corneal neovascularization (CorNV) [266]. This system integrates anti‐inflammatory and anti‐VEGF properties with chemoexcited photodynamic therapy (PDT) by loading CPPO and Ce6 into the phospholipid bilayer and anchoring ranibizumab (Rani) to the surface via ROS‐cleavable thioketal (TK) bonds. In this NCCR architecture, the nanovesicles leverage intrinsic chemokine receptors to home into inflammatory lesions, while the ROS‐responsive linkage ensures localized Rani release at the neovascular site, thereby effectively inhibiting vascular endothelial cell activation.

Furthermore, pseudo‐neutrophil cytokine sponges exploit the dense repertoire of endogenous cytokine receptors on the neutrophil membrane to serve as “decoys” that capture and neutralize proinflammatory factors, such as TNF‐α, IL‐1β, and IL‐6, to mitigate cytokine storms or chronic inflammation. A representative study introduced immune‐defensive hydrogel microspheres (GM@T‐NNPs) for intervertebral disc degeneration (IVDD) therapy [267]. These microspheres are surface‐grafted with neutrophil‐membrane‐camouflaged NPs (T‐NNPs) that inherit essential cytokine receptors from their parent cells. Functioning as a broad‐spectrum antagonist, this composite system effectively traps circulating proinflammatory cytokines, dampens the inflammatory cascade, and promotes the regeneration of the nucleus pulposus by restoring extracellular matrix homeostasis.

In summary, through strategic ligand modification, responsive linkages, and the implementation of “cytokine sponges,” these platforms not only inherit the innate inflammatory homing and immunomodulatory properties of neutrophils but also achieve multidimensional integration of therapeutic functions with spatiotemporally controlled release. Ranging from localized precision intervention in CorNV to the systemic remodeling of the inflammatory microenvironment in IVDD, these collective findings demonstrate a novel paradigm of precision therapy that is flexibly programmable and possesses both active targeting capabilities and pathological adaptability. As membrane engineering technology increasingly converges with synthetic biology, neutrophil‐membrane based‐biomimetic systems are poised to facilitate a therapeutic evolution from “passive camouflage” to “active regulation” across a broader spectrum of inflammation‐related disorders.

### 5.3 Neutrophil‐Derived Vesicles and Exosomes

The inherent advantages of exosomes derived from neutrophils for therapeutic application are significant: they exhibit low immunogenicity, high stability in the extracellular space for up to 5 days, and a superior ability to penetrate deep into tissues and cross endothelial barriers. Furthermore, their surface protein profile often includes “do not eat me” signals such as CD47, CD24, and CD31, which protect them from premature efferocytotic clearance by macrophages, thereby prolonging their circulation and functional efficacy compared with intact parent cells [268]. For therapeutic applications, cargo can be loaded into nEVs through either passive or active approaches. Passive loading involves direct incubation of isolated exosomes with therapeutic cargos or incubation of donor neutrophils with the desired cargo prior to vesicle isolation. Active loading strategies, including extrusion, sonication, freeze–thaw cycling, and electroporation, facilitate cargo incorporation by transiently disrupting the vesicular membrane. These approaches provide complementary options for loading diverse therapeutic cargos while maintaining the structural and functional properties of nEVs [297].

Recent high‐impact studies have elucidated the mechanisms by which these vesicles coordinate the resolution of inflammation. Hsu et al. developed a research framework identifying large aging‐neutrophil‐derived vesicles (LAND‐Vs) as pivotal regulators of the complement system [271]. Their design utilized Staphylococcus aureus‐induced pneumonia models and neutrophil‐specific CD55 conditional knockout mice to characterize the biogenesis and function of these >1 µm particles. The study highlights that during the neutrophil aging process, CD55 (decay‐accelerating factor) polarizes into ordered lipid raft domains, leading to the energy‐dependent budding of CD55^+^ LAND‐Vs; these vesicles then act as potent, cell‐free inhibitors of the complement 3 convertase, effectively reducing excessive neutrophil recruitment and alleviating tissue damage in acute infection. Complementing this, Kang et al. designed an investigation into the role of neutrophil‐to‐macrophage communication via nEVs in the context of cytokine storm syndrome [79]. By employing poly I:C and lipopolysaccharide‐induced models alongside intravital imaging, they tracked the generation of vesicles and their capture by resident macrophages in the liver and spleen. A primary highlight of their work was the discovery that nEVs deliver miR‐27a‐3p to recipient macrophages, which suppresses the Suclg1 gene an enzyme responsible for itaconate catabolism thereby inducing the accumulation of the anti‐inflammatory metabolite itaconate and reprogramming macrophages toward a proresolving phenotype. The significance of nEV integrity is further underscored in chronic inflammatory pathologies. Yu et al. characterized the quantitative and qualitative defects of PMN‐derived exosomes in Behçet’ s disease (BD) [274]. Their study design compared active and inactive BD patients with healthy controls, utilizing miRNA sequencing to profile the exosomal cargo. The research highlights that BD PMN exosomes are not only reduced in quantity but are also significantly depleted of miR‐122‐5p. This deficiency results in a failure to target and degrade the transcription factor IRF5 in macrophages, leading to the overactivation of TLR4 signaling and IFN‐β autocrine loops, which ultimately drives the systemic vasculitis characteristic of the disease.

Collectively, these findings establish nEVs as sophisticated delivery vehicles that utilize specific protein and RNA cargo to exert systemic control over innate immune homeostasis.

### 5.4 Comparative Evaluation and Selection Criteria of Neutrophil‐Based Delivery Platforms

By integrating the biological properties of neutrophils with engineering strategies, these platforms have been investigated for targeted therapy in sepsis, ALI, neurological disorders, and cancer. Although they share inflammatory‐site targeting and responsiveness to pathological microenvironments, their properties differ in ways that determine their suitability for specific applications.

Living neutrophil carriers retain intrinsic chemotaxis, endothelial transmigration, and responsiveness to inflammatory signals, enabling active accumulation at disease sites. They may therefore be particularly useful for acute inflammation, I/R injury, and tumors with strong neutrophil recruitment. However, their translation is constrained by cell isolation, cargo loading, viability, functional variability, and large‐scale manufacturing.

Neutrophil membrane‐coated NPs retain key membrane proteins involved in inflammatory homing while avoiding the challenges of maintaining living cells. They also offer greater flexibility in cargo selection and improved physicochemical stability. However, their targeting depends on the preservation and orientation of functional membrane components, and their passive nature may limit adaptation to dynamic pathological cues. Species differences may further complicate their translation. Although these systems have shown BBB penetration and therapeutic effects in mouse models of stroke and glioma, differences between murine and human neutrophils in receptor expression, adhesion molecules, chemokine responses, and activation states may affect endothelial interactions, transendothelial migration, and tissue targeting. Further validation using human neutrophil membranes and human BBB models is therefore needed [298].

Neutrophil‐derived vesicles and exosomes provide a cell‐free alternative that retains endogenous targeting properties while offering potential advantages in biosafety and manufacturing. However, limited cargo loading, production yield, purification standardization, and batch‐to‐batch variability remain important challenges.

Compared with other cell‐based delivery systems, neutrophils offer a distinct combination of active chemotaxis, inflammatory tropism, and barrier‐crossing capacity. Macrophage‐based carriers provide strong phagocytic capacity and tissue retention but are susceptible to microenvironment‐dependent polarization. Red blood cell‐based carriers offer prolonged circulation and good biocompatibility but limited active targeting, whereas platelet‐based carriers are particularly suited to damaged vasculature and tumor‐associated vascular interactions. Neutrophils may therefore be advantageous when active recruitment to inflammatory lesions and barrier penetration are required, although their short lifespan and susceptibility to pathological activation and functional reprogramming remain important limitations [299, 300].

Table 6 compares the three neutrophil‐based delivery platforms in terms of targeting mechanisms, cargo‐loading flexibility, production complexity, immunogenicity, stability, and translational feasibility. Overall, no single cell‐based delivery platform is universally superior. Platform selection should be guided by disease characteristics, therapeutic objectives, and translational requirements. Neutrophil‐based systems may be preferable when active inflammatory recruitment and barrier penetration are prioritized, whereas other cell‐based carriers may be more suitable when prolonged circulation, tissue retention, or vascular adhesion is required.

**TABLE 6:** Characteristics, advantages, and translational challenges of various neutrophil‐based drug delivery strategies.

Feature	Neutrophils as living cell drug delivery systems	Neutrophil membrane‐coated nanoparticles	Neutrophil‐derived vesicles and exosomes
Cargo loading	Ex vivo loading in situ neutrophil hijacking [285]	Encapsulation/physical entrapment [301] Hydrophobic interaction [302] Electrostatic interaction Chemical conjugation [285]	Passive loading: incubate the exosomes directly with the drug; incubate the drug with the exosome‐donor cells Active loading: extrusion; sonication; freeze–thaw cycle method; electroporation [297]
In vivo advantages	Active migration Inflammatory tropism Tissue penetration [303] Pathological cue responsiveness	Improved biocompatibility Enhanced tissue targeting Reduced immune clearance [298]	Natural targeting ability Protection of cargo from degradation Efficient drug delivery to diseased tissues [304]
Typical applications	Inflammation; infection; ischemia/reperfusion injury; solid tumors	Cancer; inflammation; infection; tissue‐targeted delivery [305]	Cancer; inflammation; tissue injury; biomolecule delivery
Preclinical limitations	Variable phenotype Limited cargo loading Short lifespan, cargo leakage Unpredictable in vivo behavior [306]	Variable coating efficiency Incomplete membrane protein preservation/orientation Uncertain long‐term fate	Low/variable yield Heterogeneous composition Limited loading efficiency Incomplete understanding of in vivo fate [307]
Clinical translation barriers	Donor/source variability Activation‐state control Potency assays, GMP manufacturing Cryopreservation Batch consistency [283]	Scalable membrane preparation Coating standardization Quality control Reproducibility Long‐term safety	Scalable production Purification Characterization Potency assays Loading standardization Storage Regulatory classification

## 6 Key Challenges and Technical Bottlenecks in Clinical Translation

Despite the promising potential of NDDS, their clinical translation remains challenging due to biological, engineering, manufacturing, and regulatory barriers, which vary across different neutrophil‐targeting and neutrophil‐based delivery strategies. As summarized in the upper panel of Figure 5, these challenges and potential optimization strategies are discussed from biological, platform engineering, manufacturing, quality control, and regulatory perspectives.

**FIGURE 5:**
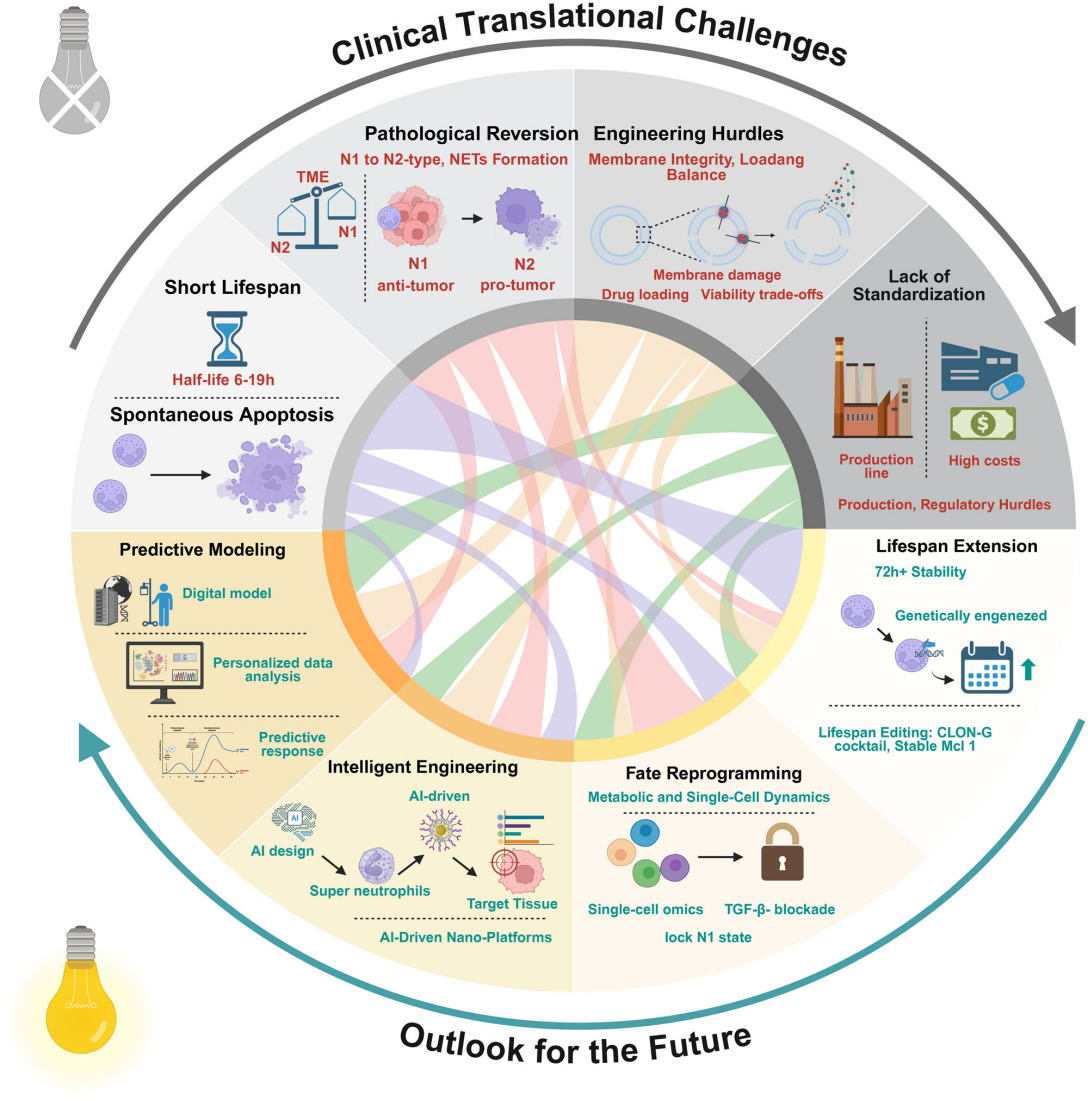
Clinical translational challenges and future directions of neutrophil‐based therapies. Upper panel: barriers to clinical translation (short lifespan, apoptosis, polarization, engineering constraints, standardization issues). Lower panel: emerging solutions (lifespan extension, reprogramming, intelligent engineering, predictive modeling) to improve precision and efficacy.

### 6.1 Strategy‐Specific Biological Constraints

The physiological properties of neutrophils provide the foundation for active targeting while also imposing important constraints on clinical translation. For broad‐spectrum neutrophil modulation, the central challenge is to suppress pathological functions without compromising essential antimicrobial activity. Global inhibition of neutrophil recruitment or activation may reduce tissue injury but also impair host defense, supporting a shift toward subset‐ or function‐selective strategies that target disease‐associated neutrophil populations or pathological effector functions while preserving basal antimicrobial activity [9, 74]. The short physiological lifespan of neutrophils remains a major obstacle for adoptive cell therapies. As terminally differentiated effector cells, neutrophils have been reported to have a circulatory half‐life of approximately 6–19 h [189, 308], providing a limited window for cell isolation, cargo loading, quality control, and reinfusion before apoptosis occurs [206]. Mechanical stress during cell processing may further accelerate functional decline and clearance. Longevity‐promoting approaches, such as the caspases–LMP–oxidant–necroptosis inhibitor plus G‐CSF (CLON‐G) cocktail, and low‐activation loading protocols may extend the ex vivo functional window and preserve neutrophil function, although their effects on in vivo performance remain to be established [309]. Autologous production reduces immunological compatibility concerns but limits scalability and introduces patient‐to‐patient variability, whereas allogeneic neutrophils may improve manufacturing flexibility but raise concerns regarding donor variability and immunological compatibility. Preservation of neutrophil chemotactic and migratory capacity is also critical for targeted delivery. Neutrophil recruitment is regulated by the CXCL12/CXCR4 and CXCL2/CXCR2 axes [87], which may become dysregulated in chronic inflammation and advanced cancer. Defects in β2 integrins or Kindlin‐3, as demonstrated in LAD syndromes, can impair transendothelial migration [310]. Such variability may reduce lesion‐specific accumulation and increase off‐target delivery, highlighting the need for functional assessment of chemotaxis and migration before administration.

### 6.2 Functional Stability and Pathological Reprogramming

A critical challenge in repurposing neutrophils as therapeutic carriers is their inherent functional plasticity, which may allow the vehicle to revert to a pathogenic role within the diseased microenvironment. This instability may reduce therapeutic predictability and introduce safety risks.

Beyond phenotypic plasticity, the clinical translation of neutrophil‐based therapies is further complicated by the dual nature of inflammation itself. Inflammation is indispensable for pathogen elimination, tissue repair, and restoration of homeostasis; however, unresolved or excessive inflammatory activation can promote chronic tissue injury and disease progression. This “inflammation paradox” indicates that therapeutic strategies should not aim to eliminate inflammatory responses completely, but rather restore the balance between protective and pathological immune functions. Similar principles have been observed in other innate immune populations, where timely transitions between inflammatory and reparative states are essential for successful tissue regeneration. For example, macrophages promote early inflammatory clearance after injury but subsequently undergo functional switching to support tissue reconstruction, indicating that stage‐specific modulation is superior to broad immune suppression [311].

The most prominent manifestation of this plasticity is the polarization of neutrophils into N1 and N2 states. Within the TME, carrier cells are exposed to high concentrations of TGF‐β and IL‐6, which can reprogram neutrophils to adopt an immunosuppressive N2 phenotype [176]. In this state, the intended delivery vehicle may paradoxically facilitate tumor progression by promoting angiogenesis and secreting factors like ARG1 that paralyze cytotoxic T‐cell responses [176]. Therefore, controlling neutrophil activation and disease‐associated functional programs before and after administration may be more appropriate than attempting to maintain a fixed phenotype.

A similar challenge applies to oxidative stress‐mediated neutrophil functions. Although ROS production represents a fundamental antimicrobial mechanism required for pathogen clearance, excessive oxidative stress can disrupt tissue homeostasis and amplify pathological inflammation. Thus, oxidative stress should be regarded as a context‐dependent regulator rather than an entirely detrimental process [312]. Therapeutic strategies targeting neutrophil oxidative responses should therefore focus on restoring redox balance and limiting pathological oxidative damage while maintaining essential host defense capacity.

Secondary injuries associated with the effector functions of neutrophils also present a significant clinical hurdle. While the release of NETs is often exploited as a stimulus‐responsive drug release mechanism, these chromatin scaffolds can induce collateral damage. Excessive NET formation has been shown to shield CTCs from immune surveillance and facilitate metastatic seeding by activating the CCDC25 receptor on malignant cells [178]. Furthermore, the explosive release of granular proteases, such as NE, can degrade healthy tissue and exacerbate SIRS. The extent of neutrophil activation, NET formation, and protease release should therefore be considered important safety parameters during platform development.

### 6.3 Engineering Optimization of Neutrophil‐Based Delivery Platforms

Beyond biological constraints, the construction of stable and functional neutrophil‐based delivery platforms presents several engineering challenges, particularly in cargo loading and membrane engineering. The trade‐off between therapeutic payload capacity and cellular viability presents a significant challenge for living neutrophil carriers. High concentrations of encapsulated drugs or NPs can trigger premature neutrophil activation and degranulation, causing cargo release before reaching the target site [216]. Furthermore, the rate of drug internalization is sensitive to cargo morphology, with rod‐shaped NPs reported to be internalized more efficiently than spherical counterparts [282]. Cargo size, morphology, surface properties, and loading conditions should therefore be optimized to maximize payload delivery while minimizing premature activation and functional impairment. The fabrication of neutrophil membrane‐coated NPs relies on the transfer of the native protein repertoire onto synthetic cores. However, mechanical extrusion or sonication can compromise membrane integrity and the orientation of critical surface receptors [313]. For instance, the adhesive potential of CD11b/CD18 complexes is contingent upon their “right‐side‐out” orientation and the preservation of higher‐order structures like lipid rafts. Therefore, optimizing membrane‐processing conditions to preserve membrane integrity and functional protein presentation remains an important engineering challenge [217]. Controlled membrane assembly, optimized extrusion or microfluidic processing, and membrane engineering may improve coating uniformity and receptor accessibility. In addition, optimization of particle surface properties may reduce protein‐corona‐mediated masking of membrane‐derived targeting proteins in vivo. Quantitative assessment of coating completeness, membrane protein orientation, and receptor accessibility should therefore be incorporated into platform‐specific quality control.

For neutrophil‐derived vesicles and exosomes, improving production yield, cargo loading, purification, and batch consistency remains essential for scalable manufacturing. Particle size, morphology, surface chemistry, and membrane composition should be systematically optimized according to their effects on cellular uptake, intracellular trafficking, and cargo release [314].

### 6.4 Manufacturing and Quality‐Control Requirements

The transition of neutrophil‐based platforms from successful animal models to human patients is hindered by the lack of standardized manufacturing and potency assessment. Importantly, quality attributes should be defined according to the specific delivery strategy rather than relying on viability or conventional physicochemical measurements alone.

For living neutrophil carriers, critical quality attributes (CQAs) should include viability, activation state, chemotactic responsiveness, migration capacity, cargo retention, and NET formation [7]. For neutrophil membrane‐coated NPs, relevant attributes include particle size, membrane coverage, membrane protein composition and orientation, receptor accessibility, cargo retention, and targeting activity [298]. For neutrophil‐derived vesicles and exosomes, particle characteristics, cargo content, purity, membrane markers, and batch‐to‐batch consistency are particularly important. Establishing standardized potency assays that link these quality attributes to functional delivery performance will be essential for reproducible manufacturing and clinical translation.

The logistical complexity and high cost of production represent another significant hurdle. Most current neutrophil‐based delivery systems utilize an autologous approach, in which cells are harvested from and reintroduced into the same patient. Although this strategy may reduce immunological compatibility concerns, it requires individualized manufacturing and limits economies of scale. Allogeneic neutrophils may improve manufacturing flexibility but raise concerns regarding donor‐to‐donor variability, immunological compatibility, and preservation of physiological functions. Moreover, the short lifespan of neutrophils creates additional challenges for ex vivo manipulation, storage, and transportation [315]. Scalable and GMP‐compatible manufacturing processes will therefore be essential for improving product consistency and clinical feasibility.

### 6.5 Regulatory and Clinical Translation

Regulatory categorization remains an important challenge because neutrophil‐based products may involve features of cell‐based therapies, biological products, conventional drugs, or combination products, depending on their composition and intended use. Regulatory expectations also differ across major jurisdictions. In the USA, living neutrophil products are likely to be evaluated within frameworks applicable to cellular or biological products, with emphasis on manufacturing control, product characterization, and potency. In the European Union, cell‐based products may fall within the advanced therapy medicinal product framework, with specific quality, nonclinical, and clinical requirements. In China, NMPA/CDE has established technical guidance for cell‐based products and nanomedicines, including quality control and nonclinical evaluation. These differences highlight the need to define platform‐specific CQAs and regulatory strategies at an early stage of development.

Finally, a substantial translational gap remains between the performance of these vehicles in preclinical models and their behavior in heterogeneous human diseases. Preclinical studies often rely on simplified or immunocompromised models that may not fully reproduce the complex vascular, immune, and metabolic environments encountered in patients. Clinically relevant disease models, standardized potency and safety assessments, and scalable GMP‐compatible manufacturing will therefore be important for determining whether therapeutic functions observed in preclinical studies can be reproduced with sufficient consistency and safety in humans.

## 7 Outlook for the Future: Toward Intelligent Regulation and Clinical Translation

Future advances in neutrophil‐based therapies will require progress at three complementary levels: resolving disease‐specific neutrophil states, developing context‐responsive engineering platforms, and selectively reprogramming pathogenic neutrophil functions. As summarized in the lower panel of Figure 5, single‐cell and multiomic approaches may define functionally relevant neutrophil states and their regulatory mechanisms; intelligent engineering may enable spatiotemporally controlled targeting and delivery; and phenotypic reprogramming may provide a means to suppress pathological functions while preserving essential host defense. The following three directions therefore highlight not only emerging opportunities but also key unresolved questions that should guide future investigation.

### 7.1 Single‐Cell Omics for Resolving Neutrophil Heterogeneity and Disease‐Specific States

A paramount objective for forthcoming research is to define how molecular heterogeneity translates into distinct neutrophil functions across disease contexts. While this review has detailed the reliance on anaerobic glycolysis during recruitment, the molecular triggers governing the transition from N1 phenotypes to N2 states characterized by FATP2‐mediated lipid accumulation remain only partially understood. A definitive instance of this metabolic hijacking was identified by Veglia et al., who demonstrated that the exclusive upregulation of FATP2 in tumor‐infiltrating neutrophils facilitates the uptake of arachidonic acid and the subsequent synthesis of PGE2, thereby paralyzing T‐cell responses [3]. A key unresolved question is whether transcriptionally defined neutrophil states represent stable functional subsets or transient states imposed by specific tissue microenvironments. Future investigations should therefore integrate single‐cell transcriptomics with epigenomic, metabolic, spatial, and functional analyses to determine which molecular signatures are causally linked to antimicrobial defense, tissue injury, immune suppression, or therapeutic responsiveness. An important challenge is to distinguish descriptive molecular signatures from functionally actionable targets. This will require experimental validation of candidate states using lineage tracing, perturbation approaches, and functional assays rather than relying solely on clustering‐based classification. Beyond phenotypic classification, rewriting the metabolic logic of these cells via the stabilization of Mcl‐1 or the upregulation of glucose transporters (GLUT1/3) may provide potential strategies to modulate neutrophil survival and function. Recent studies have reported that a pharmacological cocktail named CLON‐G, targeting caspases, lysosomal membrane permeabilization, and necroptosis, can stabilize Mcl‐1 and extend the ex vivo survival window of neutrophils to over 72 h and up to 5 days without compromising their functional integrity, providing a potential window for genetic engineering and adoptive transfer. Furthermore, GLUT1 has been shown to be indispensable for the acquisition of tissue‐damaging effector functions in models of antibody‐mediated glomerulonephritis, identifying metabolic transporters as potential tunable regulators of inflammation. Neutrophil heterogeneity is also temporal rather than purely spatial or molecular. Elucidating the “neutrophil clock,” including the circadian‐regulated interplay between CXCR2 and CXCR4 signaling, may be essential for optimizing the timing of intervention, particularly in hyperacute conditions. Recent evidence in myocardial infarction and IS has revealed that cardiac damage peaks when neutrophils are recruited during specific “Zeitgeber” phases, such as ZT13 in mice, driven by diurnal oscillations in CXCR2 expression. Notably, circadian checkpoints mediated by CXCR4 agonism have been reported to shift neutrophils toward a protective night‐like state, concentrating them at lesion sites while minimizing collateral tissue injury. Future studies should determine whether such temporal signatures are sufficiently conserved across species and disease settings to support clinically actionable chronotherapeutic strategies.

### 7.2 Intelligent Engineering for Context‐Responsive Neutrophil‐Based Therapies

A major engineering challenge is to move neutrophil‐based platforms from passive targeting toward context‐responsive and quantitatively controllable systems. Although neutrophil‐derived carriers, membrane‐coated NPs, and living‐cell delivery systems can exploit intrinsic inflammatory tropism, precise control over targeting, cargo loading, release, and cellular function remains difficult. Key unresolved questions include whether engineered systems can selectively recognize pathological neutrophil states without interfering with protective neutrophils, and whether cargo release can be dynamically controlled according to the spatial and temporal characteristics of the disease microenvironment. Digital and intelligent engineering may help address this gap by integrating patient stratification, functional prediction, and rational design of neutrophil‐based delivery systems. Digital and intelligent engineering may help address these challenges by integrating patient stratification, functional prediction, and rational design of neutrophil‐based delivery systems. A foundational pillar of this transition is the implementation of interpretable computational models for patient stratification. Traditional biomarkers often fail to capture the systemic inflammatory state that dictates treatment success; however, the Logistic Regression‐Based Immunotherapy‐Response Score represents an emerging approach for integrating clinical variables such as age, tumor mutational burden, and the peripheral NLR to identify patients who may respond to immunotherapy [316, 317]. Digital Twin‐GPT approaches further illustrate the potential of virtual patient models to integrate longitudinal clinical information and predict disease trajectories and treatment responses [318]. Complementing computational prediction, intelligent engineering may facilitate the standardization and optimization of neutrophil‐based delivery platforms. Automated high‐throughput platforms, such as TuNa‐AI, have been developed to accelerate NP formulation and identify favorable material compositions while reducing reliance on empirical optimization. Similar data‐driven approaches could be extended to optimize membrane‐coating parameters, cargo loading, particle characteristics, and release profiles, thereby establishing quantitative relationships between engineering parameters and neutrophil function.

An important future direction is therefore the development of closed‐loop platforms that couple pathological sensing with therapeutic output. Such systems could integrate disease‐associated cues, such as ROS, proteases, hypoxia, or inflammatory mediators, with programmable cargo release or modulation of neutrophil activity. The central question is whether these platforms can achieve sufficient sensitivity and temporal precision to respond to pathological signals while avoiding premature activation or nonspecific release. Digital tools may also contribute to quantitative quality control of neutrophil‐based systems. Future platforms could integrate high‐throughput imaging, microfluidic migration assays, and real‐time functional monitoring to characterize neutrophil activation, migration, cargo loading, and release [7]. Combining these measurements with predefined CQAs may reduce donor‐ and batch‐to‐batch variability and establish more reproducible potency criteria. For membrane‐coated NPs, computational and analytical approaches could further support quantitative evaluation of membrane integrity, orientation, and functional protein presentation. A remaining challenge is to determine which functional parameters best predict therapeutic performance in vivo and can therefore serve as quantitative engineering specifications. Establishing such structure‐function relationships will be essential for moving from empirical optimization toward reproducible and controllable neutrophil‐based platforms.

### 7.3 Phenotypic Reprogramming for Precision Neutrophil Immunomodulation

Rather than broadly suppressing neutrophils, an emerging therapeutic goal is to selectively reprogram pathogenic neutrophil states while preserving essential host‐defense functions. The clinical translation of neutrophil‐based interventions is therefore increasingly departing from broad depletion strategies toward precision immunomodulation of discrete functional subsets.

In the oncology arena, TANs are now appreciated as functionally heterogeneous populations with context‐dependent activities. Experimental blockade of TGF‐β signaling has been shown to shift TANs toward an N1‐like state, enhancing cytotoxicity and reducing metastatic dissemination in murine colorectal cancer models [319, 320]. These findings underscore the therapeutic potential of reprogramming dysfunctional neutrophils rather than eliminating them indiscriminately.

However, several fundamental questions remain unresolved. Can pathogenic neutrophils be durably or reversibly converted into tissue‐protective states? Which signaling and metabolic nodes determine whether such reprogramming is stable, context dependent, or reversible? Most importantly, can pathological functions such as excessive NET formation, degranulation, and tissue‐destructive inflammation be selectively suppressed without compromising antimicrobial defense?

The dualistic biology of NETs further illustrates this challenge. While NETs function as potent antimicrobial scaffolds, their dysregulated formation contributes to secondary tissue injury across a spectrum of diseases. NETosis has been implicated in deep vein thrombosis, where NET‐DNA and histones serve as prothrombotic frameworks that augment platelet aggregation [321]; in neurodegenerative contexts such as AD, NETs exacerbate neuroinflammation and BBB disruption [322]; and in cancer, excessive NET release fosters immunosuppressive niches that facilitate metastasis [20]. Emerging nanomaterial designs incorporate DNase functionalization or histone‐binding moieties to selectively dismantle pathogenic NETs in situ, providing a potential strategy to mitigate pathological inflammation while preserving host defense. For neutrophil‐based cellular delivery systems, future engineering should also incorporate functional quality assessment beyond conventional viability measurements. Chemotactic responsiveness, transendothelial migration, deformability, and cargo‐delivery capacity may provide more informative potency attributes than viability alone [323]. Standardized assessment of activation state and donor‐dependent variability will likewise be important for maintaining reproducible carrier function.

### 7.4 Rational Combination Strategies for Integrated Neutrophil Modulation

Because neutrophil‐mediated pathology involves multiple interconnected mechanisms, combination strategies may provide greater therapeutic efficacy than single‐agent neutrophil modulation. Emerging preclinical studies have explored the integration of neutrophil regulation with immune checkpoint blockade, radiotherapy, and multimodal targeting of NET‐associated pathology.

Combining neutrophil modulation with immune checkpoint blockade may help restore immune balance in diseases characterized by both excessive inflammation and immunosuppression. For example, an artificial exosome nanodecoy (AT@NV‐PD1) was developed to simultaneously regulate pathological neutrophil fate and the PD‐1/PD‐L1 axis in sepsis. AT@NV‐PD1 delivered the cyclin‐dependent kinase inhibitor AT7519 to promote apoptosis of senescent‐like neutrophils, while membrane‐presented PD‐1 sequestered PD‐L1 and alleviated T‐cell exhaustion. This dual action reduced excessive inflammation and immunosuppression, highlighting the potential of combining neutrophil‐selective modulation with immune checkpoint blockade [324].

Biomimetic delivery can also be integrated with radiotherapy to improve the functional state of TANs. Radiotherapy can induce neutrophil recruitment but may also promote protumor N2‐like polarization and NET formation. To address this limitation, dextran‐based hydrogel microparticles (L/G@HMPs) codelivering the TLR4 agonist LPS and the PAD4 inhibitor GSK484 were developed to locally reprogram RT‐recruited TANs. The system promoted N1‐like polarization, suppressed NET formation, and prolonged neutrophil survival, thereby enhancing the antitumor efficacy of radiotherapy. This strategy illustrates how localized biomimetic delivery can convert therapy‐induced neutrophil recruitment from a potential resistance mechanism into a therapeutic opportunity [325].

For NET‐driven diseases, multimodal targeting of interconnected pathological pathways may provide broader control than single‐target intervention. Zhang and colleagues developed a neutrophil membrane‐coated multifunctional nanoscavenger incorporating ROS‐scavenging Mn_3_O_4_ nanozymes and NET‐degrading DNase‐1 for severe acute kidney injury. The platform combined inflammatory‐site targeting, ROS clearance, NET degradation, and downstream cGAS–STING pathway inhibition, thereby reducing inflammatory injury and remodeling the immune microenvironment. Together, these studies support the development of rational combination therapies that integrate neutrophil modulation, spatially controlled delivery, and complementary therapeutic mechanisms, providing a promising direction for improving the efficacy and translational potential of neutrophil‐based therapies [326].

### 7.5 Safety Evaluation and Monitoring of Neutrophil‐Targeted Therapies

The therapeutic selectivity of neutrophil‐targeted strategies should be evaluated together with their potential effects on physiological host defense and systemic inflammation. A standardized safety monitoring framework should therefore integrate hematological, NET‐associated, and organ‐level indicators. Peripheral complete blood counts and ANCs are essential for detecting excessive neutrophil depletion, while the NLR may provide an additional indicator of systemic immune and inflammatory balance.

Circulating NET‐associated biomarkers may provide complementary information on pathological NET formation and the risk of thrombosis and inflammation. Cell‐free DNA, MPO–DNA complexes, and citrullinated histone H3 can be monitored to evaluate changes in NET burden, particularly for therapies targeting neutrophil recruitment, activation, or NETosis [327]. These measurements may be particularly informative when neutrophil‐targeted interventions are combined with immunotherapy or other treatments associated with inflammatory or thrombotic complications [328].

Organ‐level monitoring is also required to distinguish therapeutic effects from systemic inflammatory toxicity. Serum inflammatory markers such as CRP, IL‐6, and TNF‐α, together with organ‐specific indicators such as aminotransferases and creatinine, can provide complementary evidence of hepatic, renal, or systemic inflammatory injury [329]. Future clinical studies should therefore incorporate serial monitoring of ANC/NLR, circulating NET markers, and organ injury indicators, together with infection surveillance and coagulation assessment when appropriate. Such multidimensional safety profiling may help define the therapeutic window of neutrophil‐targeted interventions while ensuring preservation of essential antimicrobial functions.

## 8 Conclusion

Neutrophils are increasingly recognized as key regulators of immune homeostasis and disease progression rather than merely short‐lived antimicrobial cells. Their ability to rapidly respond to pathological signals, migrate across biological barriers, and undergo functional reprogramming makes them both important therapeutic targets and potential therapeutic vehicles. However, their functional plasticity also creates a central therapeutic dilemma. The same neutrophil responses that protect against infection can contribute to tissue injury, chronic inflammation, and tumor progression. Thus, effective neutrophil‐based therapies should account for disease context and functional heterogeneity rather than treating neutrophils as a uniform population.

Current therapeutic strategies are gradually moving from broad inhibition toward more selective modulation of neutrophil recruitment, signaling, and effector functions. Targets such as CXCR1/2, C5aR1, NE, and NETosis‐associated pathways illustrate this transition. However, pathway‐specific targeting alone may not fully resolve the challenge of distinguishing pathogenic from protective neutrophil responses. In our view, future strategies should integrate neutrophil heterogeneity, disease stage, and tissue microenvironment to achieve more precise regulation. Rather than simply suppressing neutrophil activity, the goal should be to selectively inhibit pathological functions while preserving essential antimicrobial and tissue‐protective activities.

A complementary strategy is to exploit neutrophils themselves as vehicles for precision drug delivery. Living neutrophil carriers, neutrophil membrane‐coated NPs, and neutrophil‐derived vesicles each offer distinct advantages in terms of preparation, cargo loading, targeting capacity, and biological adaptability. Their comparative assessment suggests that no single platform is universally optimal. Living cell carriers provide dynamic inflammatory homing but face challenges related to cell sourcing, activation state, and manufacturing consistency. Membrane‐coated NPs offer greater engineering flexibility and controllable cargo loading, whereas neutrophil‐derived vesicles may provide favorable biocompatibility and nanoscale delivery properties. Therefore, platform selection should be guided by the pathological microenvironment, therapeutic cargo, and intended clinical application.

Despite substantial preclinical progress, several barriers must be addressed before neutrophil‐based therapies can achieve clinical translation. These include reproducible manufacturing, batch‐to‐batch consistency, functional quality control, cargo stability, safety, and the potential risks associated with excessive neutrophil activation or NET formation. Future studies should establish standardized potency assays that evaluate not only cell viability but also targeting capacity, activation state, cargo retention, and functional responses. Integration of single‐cell profiling, spatial analysis, biomaterials engineering, and disease‐specific models may further enable the development of more selective and adaptable platforms. Overall, the field is moving from broad neutrophil suppression toward precision modulation and neutrophil‐enabled delivery, with the ultimate goal of preserving beneficial neutrophil functions while selectively controlling their pathological activities.

## Author Contributions

Yibing Zhang: conceptualization, literature review and analysis, manuscript drafting, and figure preparation; Siwen Wu: literature review and analysis, and figure preparation; Yusi Wang: literature review and analysis; Die Hu: literature research and data collection; Li Yang: conceptualization, supervision of the study framework, funding acquisition, and manuscript review and editing. All authors have read and approved the final manuscript.

## Funding

This work was Supported by Sichuan Science and Technology Program (2026YFMS0001).

## Ethics Statement

The authors have nothing to report.

## Conflicts of Interest

The authors declare no conflicts of interest.

## Data Availability

The authors have nothing to report.
